# Tunable high-efficiency microwave photon detector based on a double quantum dot coupled to a superconducting high-impedance cavity

**DOI:** 10.1126/sciadv.aeb9784

**Published:** 2026-04-03

**Authors:** Fabian Oppliger, Wonjin Jang, Aldo Tarascio, Franco De Palma, Christian Reichl, Werner Wegscheider, Ville F. Maisi, Dominik Zumbühl, Pasquale Scarlino

**Affiliations:** ^1^ Hybrid Quantum Circuits Laboratory, Institute of Physics, École Polytéchnique Fédérale de Lausanne (EPFL), 1015 Lausanne, Switzerland.; ^2^ Hybrid Quantum Circuits Laboratory, Center for Quantum Science and Engineering, École Polytéchnique Fédérale de Lausanne (EPFL), 1015 Lausanne, Switzerland.; ^3^ Department of Physics, University of Basel, Klingelbergstrasse 82, 4056 Basel, Switzerland.; ^4^ Laboratory for Solid State Physics, ETH Zürich, 8093 Zürich, Switzerland.; ^5^ Quantum Center, ETH Zürich, 8093 Zürich, Switzerland.; ^6^ NanoLund and Solid State Physics, Lund University, Box 118, 22100 Lund, Sweden.

## Abstract

High-efficiency single-photon detection in the microwave domain is a key enabling technology for various quantum applications. However, the extremely low energy of microwave photons presents a fundamental challenge, preventing direct photon-to-charge conversion as achieved in optical systems using semiconductors. Here, we demonstrate continuous microwave photon detection with an efficiency approaching 70% in the single-photon regime. We use a hybrid system comprising a gate-defined double quantum dot (DQD) charge qubit in a gallium arsenide/aluminum gallium arsenide heterostructure, coupled to a high-impedance Josephson junction array cavity. We systematically optimize the hybrid architecture to maximize the detection efficiency by leveraging strong charge-photon coupling, tunable DQD tunnel rates, and the frequency tunability of both subsystems. The system efficiency is characterized over a frequency range of 3 to 5.2 gigahertz. Our results establish semiconductor-based cavity–quantum electrodynamics architectures as a scalable and versatile platform for efficient microwave photon detection, opening promising avenues for quantum microwave optics and quantum information technologies.

## Teaser

Tunable QDs in a superconducting cavity approaches ultrastrong coupling limit for practical single microwave photon detection.

## INTRODUCTION

The ability to efficiently detect single photons is fundamental to a broad range of quantum technologies, including quantum communication, cryptography, sensing, and information processing (*1*–*4*). While in the optical domain single-photon detection is a mature technology—with photodiodes, avalanche photodetectors, and superconducting nanowires achieving near-unity efficiency (*1*, *5*–*8*)—the transition to the microwave regime presents profound challenges. At gigahertz frequencies, the energy of a single photon is five orders of magnitude smaller than that of an optical photon, rendering traditional photoelectric detection mechanisms inapplicable (*9*, *10*). As a result, alternative architectures are required to realize microwave single-photon detectors capable of operating at the quantum limit.

Various strategies have been developed to address this challenge, leveraging tools from the circuit quantum electrodynamics (cQED) framework (*9*). These include superconducting qubit–based detectors that map photon absorption onto quantum state transitions (*11*–*13*), biased Josephson junctions (JJs) (*14*–*18*), parametrically driven Kerr resonators operated at criticality (*19*), and bolometric sensors based on materials such as graphene (*20*–*22*). While these approaches have demonstrated key milestones such as quantum nondemolition detection and subfemtowatt sensitivity, they are often constrained by requirements for pulsed operation, complex reset protocols, sensitivity to quasiparticles, or limited long-term stability (*11*–*17*). Moreover, continuous and passive detection of itinerant microwave photons remains elusive.

An emerging and promising approach involves hybrid semiconductor-superconductor architectures, in particular double quantum dots (DQDs) embedded in high-impedance microwave cavities (*23*–*26*). When operated in the charge qubit regime, DQDs has energy splittings in the microwave domain, allowing them to absorb cavity photons and convert them into a measurable electrical current—a mechanism analogous to that of optical photodiodes (*27*–*31*). Unlike many superconducting qubit–based detectors, DQD detectors can operate continuously, without active qubit reset or prior knowledge of photon arrival time (*27*, *29*, *30*, *32*). Their well-defined energy structure provides intrinsic frequency selectivity, and the absence of JJs in the absorption medium eliminates sensitivity to quasiparticle-induced dark counts, a key advantage for robust operation (*14*–*17*).

In recent years, pioneering experiments have demonstrated photon-assisted tunneling (PAT) in DQD-cavity hybrid systems, validating their potential as microwave photodetectors (*27*, *29*, *32*). However, the reported efficiencies have so far remained limited, largely due to the poor tunability of the semiconducting host materials used and the low impedance of the superconducting cavities used. These limitations have constrained the coupling strength and matching of photon absorption rates, necessary for optimal photoconversion. As a result, the realization of high-efficiency, continuous microwave photon detection in such systems has remained an open challenge.

In this work, we demonstrate a semiconductor DQD–based microwave photon detector operating in a fully passive and continuous mode. We report a microwave photon detection efficiency approaching 70%, marking a substantial improvement over previously reported semiconductor-based detectors (*27*, *32*). This performance is enabled by strong charge-photon coupling, made possible through the high impedance of the superconducting cavity, the in situ tunability of the gate-defined DQD tunneling rates, and the inherently low charge relaxation rate of the DQD. In addition, the frequency tunability of both the cavity and the DQD excitation energy allows for frequency-selective detection across a broad range of 3 to 5.2 GHz. To accurately quantify the detection efficiency in the single-photon regime, we perform a careful calibration of the input photon flux via ac Stark shift measurements of the charge qubit frequency (*33*). Together, these results mark a crucial step toward scalable and high-fidelity microwave photon detection in hybrid quantum systems.

## RESULTS

### Device architecture

**Fig. 1.**
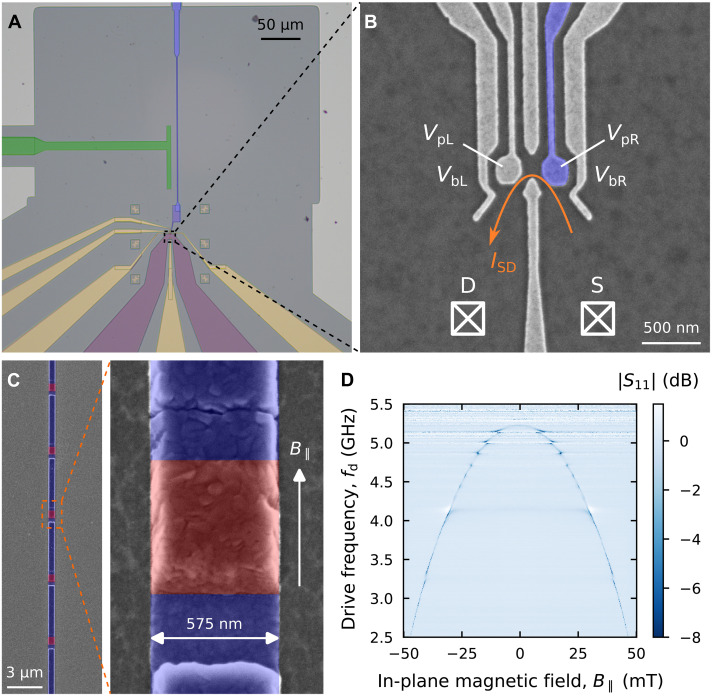
Hybrid device architecture and frequency tunability of the microwave cavity. (**A**) False-colored optical micrograph of the hybrid device with the DQD gates in yellow, the mesa region with the 2DEG in purple, the JJ array cavity in blue, and the coupling capacitor to the feedline in green. (**B**) Scanning electron micrograph of the DQD gate structure. The right plunger gate (in blue) is galvanically connected to the end of the JJ array cavity. (**C**) Scanning electron micrograph of the JJ array cavity with a zoom-in on a single junction. The regions containing the JJs are highlighted in red. (**D**) Normalized cavity reflectance $|S_{11}|$ measured at low drive power as a function of the cavity drive frequency $f_{d}$ and in-plane magnetic field $B_{∥}$ parallel to the JJ array, indicated by the white arrow in (C). The cavity resonance frequency is tuned, as the Josephson inductance *L* is modulated by the magnetic flux penetrating the JJs (*35*).

Figure 1A shows an optical micrograph of the semiconductor-superconductor hybrid cQED device built on a GaAs/AlGaAs heterostructure. A two-dimensional electron gas (2DEG) is accumulated at the top GaAs/AlGaAs interface via remote doping. To form a well-defined conductive channel and to minimize dissipation of microwave signals, we locally removed the 2DEG by etching, such that only a small mesa region remains (purple shaded region in Fig. 1A). Ohmic contacts to the 2DEG (white crossed boxes in Fig. 1B) allow to probe the source-drain current $I_{\text{SD}}$ across the DQD (see setup described in text S3). A scanning electron micrograph of a zoomed-in region denoted by the dashed box in Fig. 1A is shown in Fig. 1B. A DQD is laterally defined by Ti/Au gates patterned on top of the heterostructure (Fig. 1B). The right plunger gate, shaded in blue in Fig. 1B, is galvanically connected to a superconducting high-impedance cavity. The cavity consists of an array of *N* = 24 Al/Al_2_O_3_/Al JJs and is shown in Fig. 1C (blue shaded structure in Fig. 1A) (*34*). The other end of the cavity is shunted to ground to form a quarterwave cavity. The JJ array is fabricated by two angled evaporation steps with an oxidation step in between to form a JJ in the overlapping region of the two Al electrodes (red shaded region in Fig. 1C). Further fabrication details can be found in text S1.

A single-port feedline is capacitively coupled to the cavity (green shaded structure in Fig. 1A), allowing to probe the cavity response with a reflection measurement. The microwave photons transferred from the feedline to the cavity are absorbed by the DQD enabling photon-to-electron conversion (*27*, *32*). Thereby, it is preferable to maximize the feedline-cavity coupling rate $\kappa_{c}$ to enable efficient photon transfer, as will be detailed later. Notably, the resonance frequency $f_{c}$ of the JJ array cavity is tunable by applying a magnetic field $B_{∥}$ parallel to the JJ array (*35*) as demonstrated in Fig. 1D. At $B_{∥}=0$, the cavity resonates at $f_{c}∼5.2$ GHz, set by the total equivalent inductance (L∼36 nH), and the total stray capacitance (C∼26 fF) of the cavity, resulting in an impedance of Z∼1.2 kohms (*34*). A finite $B_{∥}$ penetrating the JJs effectively tunes *L* to induce a modulation of the resonance frequency (*35*). We attribute the spurious resonances that appear independently of $B_{∥}$ in Fig. 1D to parasitic modes arising from the device packaging (*36*), insufficient chip grounding (leading to slot modes) (*37*, *38*), and impedance mismatch introduced by the limited bandwidth of the circulators above 4.5 GHz. The latter is mitigated using a different circulator setup for high-frequency measurements (see text S3).

### Photodetection

**Fig. 2.**
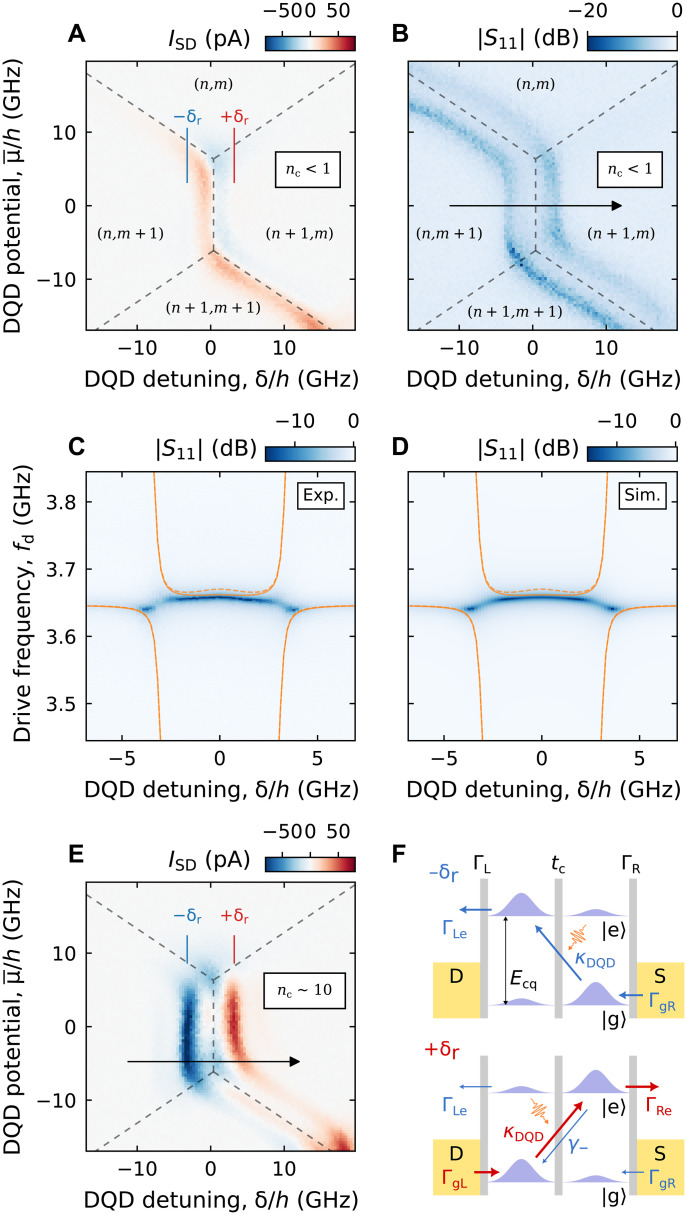
Observation of photon-induced dc current. (**A** and **B**) DQD charge stability diagram as a function of DQD detuning δ and potential $\bar{\mu }$ recorded by simultaneously monitoring (A) the DQD source-drain current $I_{\text{SD}}$ and (B) the normalized cavity reflectance $|S_{11}|$, measured with a low drive power ($n_{c}<1$). $\pm \delta_{r}$ corresponds to the DQD detuning at which the charge qubit energy matches that of the cavity photon. (n,m) represents the charge occupation numbers of the DQD. (**C**) Experimental (Exp.) $|S_{11}|$ measured as a function of drive frequency $f_{d}$ and δ, with the bare cavity resonance frequency $f_{c}=3.646$ GHz. (**D**) Simulation of $|S_{11}|$ with the parameters extracted from a numerical fit of (C) to an input-output model built from a full Rabi model Hamiltonian (see text S2). For (C) and (D), solid (dashed) orange curve corresponds to eigenspectrum derived from a full Rabi (Jaynes-Cummings) Hamiltonian. Sim., Simulation. (**E**) DQD stability diagram in the same region as (A) but measured with large cavity drive power ($n_{c}∼10$). Additional nonzero current occurs at $\delta =\pm \delta_{r}$, indicating the presence of PAT. (**F**) Schematic explaining the photoconversion process for $\delta =+\delta_{r}$ (top panel) and $\delta =-\delta_{r}$ (bottom panel). For clarity, the unwanted quenching processes $\gamma_{-}$, $\Gamma_{\text{Re}}$, and $\Gamma_{\text{gL}}$ are omitted in the top panel.

We first present the charge stability diagram of the DQD in Fig. 2A, measured by recording the source-drain current $I_{\text{SD}}$ as a function of the average chemical potential $\bar{\mu }=\left(\mu_{L}+\mu_{R}\right)/2$ and the interdot detuning $\delta =\mu_{R}-\mu_{L}$, with zero bias applied between the source and drain contacts (see Fig. 2F). Here, $\mu_{L}$ and $\mu_{R}$ denote the chemical potentials of the left and right quantum dots, respectively. $\bar{\mu }$ and δ are tuned individually by virtualizing the barrier gate voltages $V_{\text{bL}}$ and $V_{\text{bR}}$ (see Fig. 1B) (*39*). In this configuration, the DQD is tuned to have fast tunneling rates to the left ($\Gamma_{L}$) and right ($\Gamma_{R}$) reservoirs while maintaining a low interdot tunneling rate $t_{c}$ relative to the cavity frequency. (n,m) in Fig. 2A represents the charge occupation numbers of the left and right dot, respectively. In this configuration, we are able to simultaneously record $I_{\text{SD}}$ and the cavity response.

Because of the large tunnel coupling to the reservoirs, we observe clear signatures of cotunneling current between the right dot and the right reservoir. We attribute the antisymmetric currents around δ=0 to gate-voltage–induced noise, predominantly from the left plunger gate $V_{\text{pL}}$ (Fig. 1B), likely resulting from suboptimal attenuation in the microwave line (see text S4 for discussion) (*40*). However, these currents vanish at finite detunings $\delta =\pm \delta_{r}$ (indicated by the red and blue lines in Fig. 2A), where the detector operates, and therefore do not affect the photon detection measurements discussed later in this work. $\delta =\pm \delta_{r}=\pm \sqrt{{\left({\mathrm{hf}}_{c}\right)}^{2}-4t_{c}^{2}}$, corresponds to the DQD detuning at which the DQD charge qubit energy $E_{\text{cq}}=\sqrt{\delta^{2}+4t_{c}^{2}}$ is resonant with the cavity mode.

The normalized cavity reflectance $|S_{11}|$ measured at resonance is presented in Fig. 2B, with the cavity resonance frequency tuned to $f_{c}=3.646$ GHz. Initially, to avoid exciting the charge qubit, we use a low drive power such that the average intracavity photon number $n_{c}$ remains well below unity. In Fig. 2B, dips in $|S_{11}|$ appear whenever the cavity interacts with dissipative transitions in the DQD system. These features indicate an increase in the internal cavity loss rate $\kappa_{i}$, which enhances the depth of the resonance dip of an overcoupled cavity measured in reflection (*41*). Transitions involving the right QD and its reservoir, (n,m)↔(n,m+1) and (n+1,m)↔(n+1,m+1), are visible. This is consistent with the cavity being directly coupled, and therefore more sensitive, to the right plunger gate (see Fig. 1B). The observed broadening of these transitions is attributed to the large tunnel coupling between the right QD and its reservoir.

We further elucidate the hybridization between the DQD and the cavity by monitoring the cavity reflectance as a function of the charge qubit energy, varying δ along the black arrow in Fig. 2B while keeping the interdot tunnel coupling $t_{c}$ fixed. Figure 2C displays the measured cavity spectroscopy, obtained by recording the normalized reflectance $|S_{11}|$ as a function of both δ and the cavity drive frequency $f_{d}$, with the average DQD chemical potential $\bar{\mu }=0$ and average intracavity photon number $n_{c}<1$. Taking advantage of the frequency tunability of our cavity, we repeat the spectroscopy at different $f_{c}$ and fit all the collected spectra simultaneously using an input-output model that includes counterrotating terms of the full Rabi Hamiltonian (see text S2 for additional measurements and fitting details) (*42*). It is important to include these counterrotating terms because $g/\left(2\pi f_{q}\right)$ reaches values on the order of 0.1 at δ=0 (*34*, *42*). From the fit, we extract a charge-photon coupling strength of $g_{0}/2\pi ∼213.7\pm 0.3$ MHz (see table S1 for all extracted parameters). Evidently, the eigenspectrum calculated with a simple Jaynes-Cummings model (dashed curves in Fig. 2, C and D) does not closely capture the Bloch-Siegert shift near δ=0, which is present in the experiment (*42*–*44*). Instead, this shift is better reproduced by the full Rabi model (solid curves in Fig. 2, C and D), implying that the large charge-photon coupling strength substantially alters the dynamics of the hybrid system (*42*–*44*).

As discussed in more detail later, cotunneling processes play a notable role in this configuration due to the large tunnel coupling between the QDs and the reservoirs (*45*, *46*). Even when the excited state of the charge qubit remains below the reservoir Fermi level ($\bar{\mu }=0$ and δ≪U, where *U* is the interdot charging energy), electrons can tunnel out via cotunneling (*45*, *46*). As a result, the total decoherence rate extracted from the input-output model, $\Gamma_{\text{tot}}/2\pi =\left(\Gamma_{0e}+\gamma_{-}+2\gamma_{\phi }\right)/2\pi =829.3\pm 3.6$ MHz, encompasses not only the intrinsic interdot relaxation and dephasing rates ($\gamma_{-}$ and $\gamma_{\phi }$, respectively) but also the relaxation to the reservoirs $\Gamma_{0e}$ induced by cotunneling.

After having characterized our charge-photon hybrid system at low drive ($n_{c}<1$), in Fig. 2E, we increase the drive power to populate the cavity with $n_{c}∼10$ and measure the same region of the DQD stability diagram reported in Fig. 2A. Two distinct lines with positive and negative current appear exactly at $\delta =\pm \delta_{r}$ (denoted by the red and blue lines), where the qubit and the cavity are in resonance ($f_{q}=E_{\text{cq}}/h=f_{c}$), indicating the presence of photoinduced currents. As depicted in the top panel of Fig. 2F, at $\delta =-\delta_{r}$, the qubit ground (excited) state is mostly localized in the right (left) dot. An incoming photon, whose energy matches that of the qubit, can be absorbed with rate $\kappa_{\text{DQD}}=4g^{2}/\Gamma_{\text{tot}}$ and excite the qubit. Because of the spatial structure of the ground and excited state wave functions, this essentially represents a photoinduced interdot tunneling event, a process known as PAT (*47*, *48*). From the excited state, the electron can then tunnel out to the left reservoir with rate $\Gamma_{\text{Le}}=\Gamma_{L}{\text{sin}}^{2}\left(\theta /2\right)$, and the ground state can be recovered by an electron tunneling in from the right reservoir with rate $\Gamma_{\text{gR}}=\Gamma_{R}{\text{sin}}^{2}\left(\theta /2\right)$, where $\theta =\text{arccos}\left(-\delta_{r}/{\mathrm{hf}}_{c}\right)$ is the DQD mixing angle. Analogously, for $\delta =+\delta_{r}$ (bottom panel of Fig. 2F), the PAT process enables the transport of electrons from the left to the right reservoir with the rates $\Gamma_{\text{Re}}=\Gamma_{R}{\text{cos}}^{2}\left(\theta /2\right)$ and $\Gamma_{\text{gL}}=\Gamma_{L}{\text{cos}}^{2}\left(\theta /2\right)$ instead. When continuously driving the cavity, this can be observed as a positive (negative) current measured across the DQD at $+\delta_{r}$ ($-\delta_{r}$). In the bottom panel of Fig. 2F, further processes that disrupt the current across the DQD ($\gamma_{-}$, $\Gamma_{\text{Le}}$, and $\Gamma_{\text{gR}}$) are depicted, which will be discussed in more detail in the next section. While the spin degeneracy may introduce an additional factor of 2 to either $\Gamma_{\text{gR}}$ or $\Gamma_{\text{Le}}$ depending on the parity of the QD charge occupation (*32*), this factor only affects the ratio between $\Gamma_{0e}$ and $\Gamma_{g0}$ and therefore does not affect the efficiency analysis.

While photoinduced currents typically appear only at $\pm \delta_{r}$ near the charge triple points (*27*, *32*), in the DQD configuration investigated here, such currents are observed as vertical lines throughout the entire region between the QD-reservoir transitions, as shown in Fig. 2E. This stems from the aforementioned large cotunneling rate, enabled by the highly transparent tunnel barriers between the QDs and the reservoirs, as well as a relatively small interdot charging energy *U* (*46*). In contrast, for an alternative DQD configuration (see fig. S4 and text S5) measured within the same device—featuring less transparent QD-reservoir barriers and a larger *U*—cotunneling processes are substantially reduced (*46*). As a result, PAT currents are confined primarily to the vicinity of the charge triple points.

### Near-unity photon detection efficiency

**Fig. 3.**
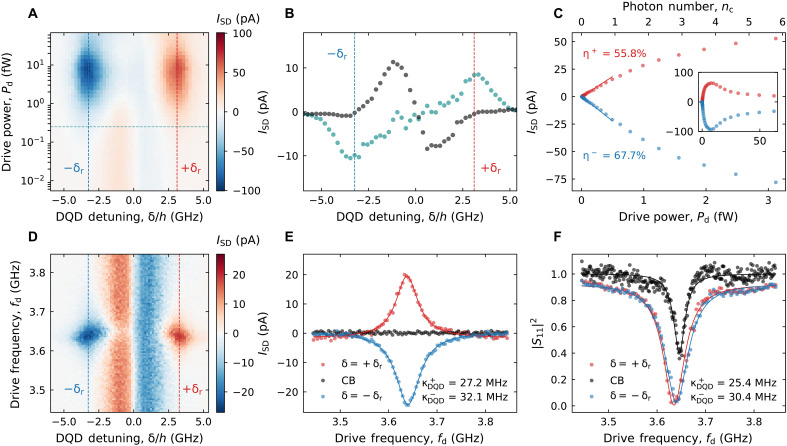
Photon detection efficiency and photon absorption rate. (**A**) DQD source-drain current $I_{\text{SD}}$ measured as a function of feedline drive power $P_{d}$ and DQD detuning δ near the charge triple points. The red (blue) dashed line indicates where $\delta =+\delta_{r}$ ($\delta =-\delta_{r}$). (**B**) The black dots denote the horizontal line cut of (A) at low $P_{d}<0.01$ fW. The cyan dots represent the effective photocurrent at finite $P_{d}$ obtained from a line cut of (A) at the cyan dashed line and subtracting the current at low $P_{d}$ (black dots). The photocurrent features clear extrema at $\delta =\pm \delta_{r}$ (red and blue dashed lines), demonstrating the PAT process. (**C**) Vertical line cuts of (A) at $\delta =\pm \delta_{r}$ for $P_{d}<3.5$ fW. A linear fit at low power ($0<n_{c}<1$) yields a photon detection efficiency of $\eta =I_{\text{SD}}/e\dot{N}=55.8\pm 4.0\%\left(67.7\pm 4.8\%\right)$ for $+\delta_{r}$ ($-\delta_{r}$). The average intracavity photon number $n_{c}$ is indicated in the top axis. The inset shows the same line cuts for the full measured range in $P_{d}$. (**D**) Measured $I_{\text{SD}}$ as a function of cavity drive frequency $f_{d}$ and δ at $n_{c}∼1$. (**E**) Line cut of (D) at $\delta =\pm \delta_{r}$ and in Coulomb blockade (CB). From a Lorentzian fit, represented by solid lines, we extract the total linewidth $\kappa_{\text{tot}}=\kappa +\kappa_{\text{DQD}}$. Together with the bare cavity linewidth κ=28.1±0.3 MHz, we obtain the DQD photon absorption rates $\kappa_{\text{DQD}}^{+}=27.2\pm 1.3$ MHz and $\kappa_{\text{DQD}}^{-}=32.1\pm 1.5$ MHz for $+\delta_{r}$ and $-\delta_{r}$, respectively. (**F**) Normalized cavity reflection ${|S_{11}|}^{2}$ as a function of $f_{d}$, with the DQD in CB and at $\delta =\pm \delta_{r}$, measured simultaneously with (E). The solid lines represent a fit to the input-output model in eq. S3, from which we extract $\kappa_{\text{DQD}}^{+}=25.4\pm 3.0$ MHz and $\kappa_{\text{DQD}}^{-}=30.4\pm 2.9$ MHz.

To quantify the performance of this device as a photodetector, we measure the photon detection efficiency $\eta =I_{\text{SD}}/e\dot{N}$, where $\dot{N}=P_{d}/{\mathrm{hf}}_{c}$ is the photon flux into the cavity feedline and $P_{d}$ is the feedline drive power at the coupling capacitor of the cavity port. $P_{d}$ is calibrated by measuring the ac Stark shift (*33*) of the charge qubit frequency (see text S6 for the full calibration procedure). In Fig. 3A, we show the measured $I_{\text{SD}}$ as a function of $P_{d}$ while varying the DQD detuning δ across the interdot transition in Fig. 2E close to the charge triple point (indicated by the black arrow). The black dots in Fig. 3B shows a horizontal line cut from Fig. 3A obtained at low $P_{d}<0.01$ fW representing the dark current of this specific configuration. The cyan dots in Fig. 3B represent a line cut at finite power after subtracting this dark current, indicated by the cyan dashed line in Fig. 3A. The photocurrent features clear extrema at $\delta =\pm \delta_{r}$ (red and blue dashed lines in Fig. 3B), demonstrating the PAT process. Figure. 3C shows vertical line cuts of Fig. 3A at $\delta =\pm \delta_{r}$ after subtracting the dark current at $\pm \delta_{r}$. For low $P_{d}$ ($n_{c}<1$), the PAT current $I_{\text{SD}}$ increases linearly, as expected from the master equation model of the system (*27*, *29*). For $n_{c}>1$, nonlinear effects due to multiphoton processes (*27*, *29*, *32*) and the self-Kerr nonlinearity of the JJ array cavity (see text S7) (*49*) begin to play a substantial role, hindering the photoconversion process. A linear fit to the slope in the range of $0<n_{c}<1$ yields an efficiency of $\eta^{+}=55.0\pm 4.0\%$ and $\eta^{-}=67.7\pm 4.8\%$ at $+\delta_{r}$ and $-\delta_{r}$, respectively.

We now fix $P_{d}$ and investigate the spectral response of the photodetector, by measuring $I_{\text{SD}}$ as a function of δ and the drive frequency $f_{d}$ at $n_{c}∼1$, as shown in Fig. 3D. As expected from theory (*27*), the photocurrent is maximized when driving at resonance, i.e., $f_{d}=f_{q}=f_{c}$, which only occurs at $\delta =\pm \delta_{r}$. Around these points, the model predicts a Lorentzian line shape with a linewidth of $\kappa_{\text{tot}}=\kappa +\kappa_{\text{DQD}}$, where $\kappa =\kappa_{c}+\kappa_{i}$ is the total bare resonator linewidth and $\kappa_{\text{DQD}}=4g^{2}/\Gamma_{\text{tot}}$ can be interpreted as the effective photon absorption rate of the DQD (*27*, *28*, *32*). Figure 3E shows a Lorentzian fit to a cut from Fig. 3D at $+\delta_{r}$ ($-\delta_{r}$), which yields $\kappa_{\text{DQD}}^{+}=27.3\pm 1.5$ MHz ($\kappa_{\text{DQD}}^{-}=32.1\pm 1.3$ MHz). Another cut at large detuning $\delta >\delta_{r}$ (black dots in Fig. 3E), for which the DQD is well in Coulomb blockade (CB), confirms that the photon absorption process is quenched when the two systems are far detuned. Alternatively, we can investigate the photon absorption by looking directly at the cavity signal instead. In Fig. 3F, we report the normalized cavity reflectance ${|S_{11}|}^{2}$ at $\delta =\pm \delta_{r}$ and in CB, measured simultaneously with the $I_{\text{SD}}$ shown in Fig. 3E. Here, the photon absorption by the DQD can be observed as an additional broadening of the resonance dip. Fitting the input-output model in eq. S3 to the measured data and subtracting the bare cavity linewidth from the $\kappa_{\text{tot}}$ yields $\kappa_{\text{DQD}}^{+}=25.4\pm 3.0$ MHz and $\kappa_{\text{DQD}}^{-}=30.4\pm 2.9$ MHz, which are close to the values previously extracted. This further confirms that the measured current through the DQD is indeed induced by absorbing photons from the cavity.

To understand what limits the photon detection efficiency from reaching unity in the current device, we use a master equation model to describe the induced photocurrent of such a hybrid system (*27*, *28*). In the low-drive limit, the induced photocurrent can be described with a linear dependence on the incoming photon flux $\dot{N}=P_{d}/{\mathrm{hf}}_{c}$ as follows

$$I_{\text{SD}}/e=\dot{N}\frac{\kappa_{c}}{\kappa }\frac{4\kappa_{\text{DQD}}\kappa }{{\left(\kappa_{\text{DQD}}+\kappa \right)}^{2}}\frac{\Gamma_{0e}}{\Gamma_{0e}+\gamma_{-}}D$$ (1)

This equation can be broken into four terms, each of which can reach values between 0 and 1. The first term describes how efficiently photons can enter from the feedline into the cavity before the photons are lost to the environment with the internal loss rate $\kappa_{i}$. Here, $\kappa_{c}/\kappa ∼0.817$ at $f_{c}=3.646$ GHz. The second term, which compares the photon loss rate κ of the bare cavity with the effective absorption rate $\kappa_{\text{DQD}}$ of the DQD, is maximized when these two rates are equal (*27*, *28*). With κ=28.1 MHz and $\kappa_{\text{DQD}}=32.1$ MHz, this term is very close to unity, $\frac{4\kappa_{\text{DQD}}\kappa }{{\left(\kappa_{\text{DQD}}+\kappa \right)}^{2}}∼0.9997$, indicating that the two systems are well matched. The third term compares the interdot relaxation rate $\gamma_{-}$ with $\Gamma_{0e}=\Gamma_{\text{Le}}+\Gamma_{\text{Re}}$ because an excited electron can only result in a dc current if it tunnels out to the reservoir before relaxing back to the ground state.

From the cavity spectroscopy near $\bar{\mu }∼0$ presented in Fig. 2C, it is not possible to estimate the interdot relaxation rate $\gamma_{-}$ because cotunneling processes contribute to the extracted decoherence rate $\Gamma_{\text{tot}}/2\pi ∼829.3\text{MHz}$ (*23*–*25*). Therefore, it is not possible to individually determine $\Gamma_{0e}$ from the presented measurements. Instead, we provide a lower bound for $\Gamma_{0e}$ in the regime where we operate the photodetector. In this regime, near the charge triple point ($\bar{\mu }<0$), $\Gamma_{\text{tot}}$ can be estimated using the relation $\Gamma_{\text{tot}}/2\pi =4g^{2}/2{\pi \kappa }_{\text{DQD}}^{-}=$ 1315.5 MHz. Assuming that the intrinsic DQD relaxation ($\gamma_{-}$) and dephasing ($\gamma_{\phi }$) rates remain unchanged at $\bar{\mu }=0$ and at the charge triple point, the observed increase in $\Gamma_{\text{tot}}$ arises from a change in $\Gamma_{0e}$. Thus, the difference in total rates sets a lower bound on $\Gamma_{0e}$, resulting in $\Gamma_{0e}/2\pi >486.2\text{MHz}$ at $\delta =-\delta_{r}$, where $\Gamma_{g0}$ is expected to be on the same order as $\Gamma_{0e}$.

The fourth term is the directivity

$$D=\frac{\Gamma_{\text{Re}}\Gamma_{\text{gL}}-\Gamma_{\text{Le}}\Gamma_{\text{gR}}}{\Gamma_{0e}\Gamma_{g0}}$$ (2)

which describes how effectively photoelectrons are tunneling from one reservoir to the other with respect to the opposite direction (*27*, *28*, *32*). Here, $\Gamma_{g0}=\Gamma_{\text{gL}}+\Gamma_{\text{gR}}$ represents the total tunneling rate from the reservoirs to the DQD ground state. Assuming equal QD-reservoir tunneling barriers on the left and right side ($\Gamma_{L}=\Gamma_{R}$), D simplifies to $\delta_{r}/{\mathrm{hf}}_{c}∼0.877$ at $f_{c}=3.646$ GHz (*27*, *28*, *32*). Further assuming $\frac{\Gamma_{0e}}{\Gamma_{0e}+\gamma_{-}}=1$, we estimate a total photon detection efficiency of $\eta_{\text{calc}}∼71.3\%$, which is very close to the measured efficiency $\eta^{-}∼67.7\%$, a value that would be obtained with $\frac{\Gamma_{0e}}{\Gamma_{0e}+\gamma_{-}}∼0.955$. This implies that relaxation of the excited state is dominated by tunneling to the reservoirs ($\Gamma_{0e}/2\pi >$ 486.2 MHz), while interdot relaxation plays only a minor role in determining the photon detection efficiency. This finding is consistent with prior studies of $\gamma_{-}$ in similar GaAs DQD devices (*23*, *34*).

The observed asymmetry in detection efficiency for positive and negative δ is attributed to unequal tunnel couplings $\Gamma_{L}$ and $\Gamma_{R}$, resulting in different values of $\Gamma_{0e}$ depending on the detuning polarity. Although such asymmetry generally reduces the directivity D and thus η, the fact that $\eta \approx \eta_{\text{calc}}$ suggests that the asymmetry is relatively minor in our device. Therefore, the dominant factors currently limiting the efficiency from reaching unity are the coupling of the cavity to the feedline $\kappa_{c}$ and the finite directivity D. The latter could be improved by either reducing the interdot tunnel coupling $t_{c}$ or increasing the cavity frequency $f_{c}$. In text S8, we demonstrate that detection efficiencies exceeding 90% are within reach by engineering devices with lower $t_{c}$ and larger $\kappa_{c}$, which are feasible in a second generation of devices.

### Frequency tunability

**Fig. 4.**
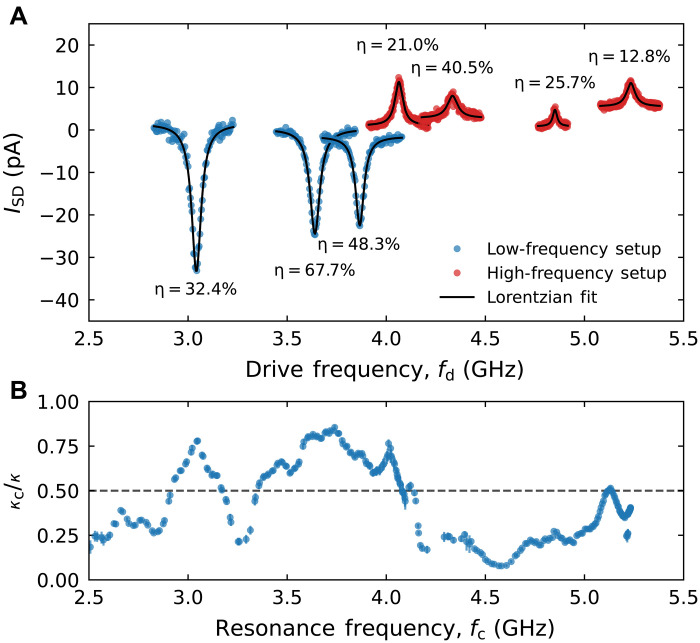
Frequency tunability of the photodetector. (**A**) DQD source-drain current $I_{\text{SD}}$ as a function of cavity drive frequency $f_{d}$ measured for different cavity resonance frequencies $f_{c}$, with a constant drive power at the rf instrument (see fig. S2). The indicated efficiency is obtained by calibrating the input losses for each $f_{c}$. The blue and red data points are measured in different DQD charge configurations and with different measurement output lines. (**B**) The ratio of the cavity-feedline coupling rate over the total loss rate of the bare cavity, $\kappa_{c}/\kappa$, as a function of $f_{c}$, obtained from fitting the cavity magnetospectroscopy in Fig. 1D (for $f_{c}<4.2$ GHz) and another similar measurement (for $f_{c}>4.2$ GHz). The dashed line represents the condition above which the cavity is overcoupled ($\kappa_{c}>\kappa_{i}$).

Exploiting the tunability of both the microwave cavity (Fig. 1D) and the DQD charge qubit, we explicitly demonstrate frequency-selective microwave photon detection. Figure 4A shows the measured photocurrent as a function of the drive frequency $f_{d}$ for different cavity resonance frequencies $f_{c}$. Because of the limited bandwidth of the microwave circulators used in our setup (see text S3), the photocurrent data marked with blue and red dots are acquired in separate cooldowns with different measurement output lines. To determine the photon detection efficiency at each resonance frequency shown in Fig. 4A, we analyze the power dependence of the PAT current, following the procedure used in Fig. 3C. For all measurements, the drive power at the instrument output is kept constant. However, because of the presence of spurious modes visible in Fig. 1D, the resulting intracavity photon number $n_{c}$ is not uniform across different measurements. This discrepancy explains why the current peak amplitudes do not scale directly with the extracted efficiencies.

As highlighted in Eq. 1, the ratio between the external coupling rate $\kappa_{c}$ and the total cavity decay rate κ sets an upper bound on the photon detection efficiency η at a given cavity frequency. Figure 4B displays the extracted values of $\kappa_{c}/\kappa$ from a numerical fit of the bare cavity magnetospectroscopy, similar to Fig. 1D. The coupling ratio fluctuates between 0.1 and 0.85, which we primarily attribute to spurious modes coupling to the cavity that act as additional loss channels, thereby altering both $\kappa_{c}$ and the internal loss rate $\kappa_{i}$. The resonance frequencies used for photoconversion efficiency characterization are selected to maximize $\kappa_{c}/\kappa$. The extracted cavity loss rates $\kappa_{c}$, $\kappa_{i}$, and κ at various frequencies are summarized in fig. S6.

## DISCUSSION

As we show in text S8, the photon detection efficiency η is sensitive to changes in several system parameters (see Eq. 1), most notably $g_{0}$, the cavity loss rates $\kappa_{c}$ and $\kappa_{i}$, and the interdot relaxation rate $\gamma_{-}$. A large $g_{0}$ ensures that the effective coupling strength at finite DQD detuning ($g=g_{0}\cdot 2t_{c}/\sqrt{\delta^{2}+4t_{c}^{2}}$) remains large across a wide range of detuning values. This enables efficient interaction between the cavity and the DQD, allowing one to achieve a condition where the effective photon absorption rate $\kappa_{\text{DQD}}=4g^{2}/\Gamma_{\text{tot}}$ can be matched to the total cavity linewidth κ with a large $\Gamma_{0e}$. In our device, this condition is achieved, thanks to the high tunability of $\Gamma_{0e}$, and carefully engineered tunnel barriers, enabled by the gate-defined architecture. A large $\Gamma_{0e}$ also makes the detection efficiency robust against interdot relaxation, as the relaxation of the excited state is dominated by tunneling to the reservoirs rather than by internal processes. Because the charge qubit dephasing rate $\gamma_{\phi }$ does not affect the qubit population but only the resonant photon absorption rate $\kappa_{\text{DQD}}$, $\gamma_{\phi }$ is not affecting the efficiency as long as the matching condition can be achieved with $\Gamma_{0e}\gg \gamma_{-}$.

An additional term influencing the efficiency is the directivity $D∼\delta_{r}/{\mathrm{hf}}_{c}$, which compares how efficiently a photoexcited electron tunnels in one direction with respect to the other. Large detuning $\delta_{r}$ and low interdot tunnel coupling $t_{c}$ are required to maximize D; however, reducing $t_{c}$ also lowers the coupling *g*. Thus, a large intrinsic $g_{0}$ is advantageous for supporting strong interaction even at reduced $t_{c}$, enabling high directivity without compromising coupling strength.

The main limitation to the efficiency of the studied DQD-cavity photon detector stems from the ratio $\kappa_{c}/\kappa$, which quantifies how efficiently photons enter the cavity from the feedline. This ratio is ultimately limited by losses intrinsic to the cavity. However, as illustrated in fig. S8A, increasing $\kappa_{c}/\kappa$ would immediately push η beyond 80%, assuming the other parameters remain unchanged. This can be achieved by increasing the coupling capacitance or by reducing the total cavity capacitance through an appropriate redesign of the device geometry. Moreover, further reducing the charge qubit relaxation rate, e.g., by increasing the mutual capacitance between the dots (*34*), can help approach near-unity detection efficiency, as confirmed in fig. S8B.

Overall, our analysis shows that efficiencies exceeding 90% are realistically achievable in this architecture with straightforward device improvements: enhanced cavity-feedline coupling, a larger cavity resonance frequency, and a reduced qubit decoherence rate. In addition, our architecture is inherently frequency selective, not susceptible to quasiparticle poisoning (*15*, *16*), and supports broadband tunability over a range of 3 to 5.2 GHz.

Three key figures of merit of a photodetector are the dead time, the noise equivalent power (NEP), and the dynamic range. The dead time $\tau_{\text{dead}}$ corresponds to the time required for the excited DQD system to reset to its ground state, which is given by $\tau_{\text{dead}}=2\pi \left(1/\Gamma_{0e}+1/\Gamma_{g0}\right)$. In our device, $\Gamma_{0e}/2\pi$ is expected to be higher than 486 MHz, implying that $\tau_{\text{dead}}$ is on the order of a few nanoseconds (assuming $\Gamma_{0e}∼\Gamma_{g0}$). This short dead time enables continuous operation of the detector, eliminating the need for heralding, pulsed gating, or explicit reset procedures (*11*–*13*). For single-photon detectors, another important figure of merit is the dark count rate, i.e., the rate at which detection events occur without the presence of an incoming photon from the signal source. For the detector presented in this work, which measures the average photon flux rather than single photons, the dark count rate cannot be estimated unambiguously, as discussed in more detail in text S4. Therefore, the NEP represents a more meaningful figure of merit than the dark count rate. The NEP represents the minimum input power required to achieve a signal-to-noise ratio (SNR) of one for an integration time of 1 s. In our setup, where the current through the DQD is continuously monitored, the noise is proportional to the current fluctuations $\delta I_{\text{SD}}$ in the dc measurement chain, where we observe $\delta I_{\text{SD}}∼50$ fA when measured with a measurement bandwidth of B=5 Hz. With a current responsivity $R=e\eta /{\mathrm{hf}}_{c}∼45$ kA/W at $f_{c}=3.646$ GHz $\text{NEP}=\delta I_{\text{SD}}/R\sqrt{B}∼5\times 10^{-19}W/\sqrt{\text{Hz}}$, consistent with values reported for other platforms (*15*, *32*). The dynamic range $\mathrm{DR}=P_{\text{max}}/P_{\text{min}}$ is the ratio between the maximum and the minimum power that can be measured. For a given bandwidth *B*, the minimum power is given $P_{\text{min}}=\text{NEP}\sqrt{B}$. We then define $P_{\text{max}}$ as the power for which the detection efficiency is reduced to half of its maximum value. At $f_{c}=3.646$ GHz and with B=5 Hz, we obtain $P_{\text{min}}∼1.1\times 10^{-18}$ W and $P_{\text{max}}∼1.7\times 10^{-15}$ W and thus DR∼32 dB.

In this work, we demonstrate high-efficiency microwave photon detection in the single-photon regime using a hybrid semiconductor-superconductor circuit QED device, achieving detection efficiencies up to η∼70%. This device substantially outperforms previously reported DQD-based detectors (*27*, *32*) and sets a benchmark for the technology. This improvement is enabled by the combination of a large charge-photon coupling strength $g_{0}$ obtained because of the high-impedance cavity, broad tunability of both the cavity frequency and the DQD tunneling rates, and the low DQD charge relaxation rate. These features are critical for simultaneously optimizing all the processes affecting the photon detection efficiency, as expressed in Eq. 1. A more detailed comparison with the detector performance in previous works can be found in text S9.

The high detection efficiency achieved with a semiconductor DQD in this work opens possibilities in the realm of quantum technology. For instance, the ability to detect individual photons with high accuracy is central for studying photon correlations (*50*, *51*), given the fidelity of *n*-photon correlation measurements scales as $\eta^{n}$. Our detector, approaching unity efficiency, offers a practical path for measuring correlations between microwave photons—substantially reducing the experimental overhead (*52*) without the need for parametric amplification chains (*11*) and for exploring quantum thermodynamic phenomena (*53*, *54*). Further architectural improvements, such as embedding a high-bandwidth charge sensor (*29*, *55*, *56*), will enable single-shot detection of itinerant microwave photons (*18*, *30*, *57*) and facilitate integration into larger quantum photonic networks. Notably, because DQD microwave photon detectors can be built on the same substrate as QD spin qubit architectures, interfacing these detectors with spin qubits could unlock promising avenues for QD spin qubit operations (*58*, *59*) and for microwave photonics—domains that have so far been largely limited to superconducting qubit systems (*9*, *58*). While integrating a QD-based microwave photon detector could introduce challenges related to wiring density or cross-talk between the detector DQD and the qubit, we expect that such effects can be mitigated by following the architecture presented in (*58*) and spatially separating the detector from the qubits by the superconducting cavity. Our work thus establishes a clear and scalable route to robust, high-performance microwave photon detection, with immediate applications in quantum communication, quantum sensing, and microwave quantum optics.

## Supplementary Materials

### This PDF file includes:

Supplementary Text S1 to S9 Figs. S1 to S8 Tables S1 to S3 References
