# Supplementary material for: Tunable high-efficiency microwave photon detector based on a double quantum dot coupled to a superconducting high-impedance cavity
Source: Sci Adv. 2026 Apr 3;12(14):eaeb9784. doi: 10.1126/sciadv.aeb9784 (PMC13048240; doi:10.1126/sciadv.aeb9784)
Supplement: Supplementary file 1 — Supplementary Text S1 to S9 Figs. S1 to S8 Tables S1 to S3 References [file sciadv.aeb9784_sm.pdf]

Science Advances
AAAS

Supplementary Materials for

Tunable high-efficiency microwave photon detector based on a double quantum dot coupled to a superconducting high-impedance cavity

Fabian Oppliger *et al.*

Corresponding author: Pasquale Scarlino, pasquale.scarlino@epfl.ch

Sci. Adv. **12**, eaeb9784 (2026)
DOI: 10.1126/sciadv.aeb9784

This PDF file includes:

Supplementary Text S1 to S9
Figs. S1 to S8
Tables S1 to S3
References

S1 Device Fabrication

The device is fabricated on a GaAs/Al_xGa_{1-x}As heterostructure with a 2-dimensional electron gas (2DEG) at the GaAs/Al_xGa_{1-x}As interface, buried 90 nm below the surface. 195/53 nm AuGe/Pt ohmic contacts are patterned by electron-beam lithography (EBL), followed by electron-beam evaporation and lift-off. The device is then annealed in forming gas (5% H_2, 95% N_2) at 490°C for 1 min to let the metal diffuse into the heterostructure and ensure good contact with the 2DEG. The self-accumulated 2DEG is etched away with a Piranha solution of H_2SO_4 : H_2O_2 30% : H_2O (1:8:640) leaving a only a small mesa region that forms a well-defined conductive channel for the DQD. The single-layer gates are patterned in two steps by EBL, evaporation and lift-off. This ensures that the thin 2/26 nm Ti/Au gates are patched on the mesa step (~ 110 s) by a second 3/110 nm layer, that is routed out to the bonding pads. A superconducting Al layer of 120 nm is deposited using EBL, evaporation and lift-off, to form the ground plane and the feedline for the cavity in a single step. Lastly, the JJ array cavity is fabricated using the conventional Dolan-bridge double angle evaporation method. Two Al layers of 35 nm and 130 nm, respectively, are deposited at an angle of 45° with an oxidation step in between to form the tunneling barrier. This tunneling oxide is grown by filling the chamber with O_2 at a pressure of 2 Torr for 20 min (static oxidation) without breaking the vacuum. Finally, after the second Al deposition, the device is once again exposed to 10 Torr of O_2 for 10 min to form a clean oxide layer at the top interface.

S2 Input-Output Model

To model the cavity response in our hybrid system, we employ an input-output model based on a quantum Rabi model to also consider the counter-rotating term, which is non-negligible in a strongly coupled system (*42, 43, 60*). We first show the Hamiltonian which describes a general cavity-qubit hybrid system. Assuming the transverse coupling between the photon and the qubit, the Hamiltonian can be written as Eq. (S1) (*61*)

$$H_{\text{full}}/\hbar = \omega_{\text{c}} a^\dagger a + \frac{\omega_{\text{q}}}{2}\sigma_z + g(a^\dagger + a)\sigma_x, \tag{S1}$$

where, $\omega_{\text{c}} = 2\pi f_{\text{c}}$ and $\omega_{\text{q}} = 2\pi f_{\text{q}}$ are the cavity and qubit frequencies, respectively, a ($a^\dagger$) is the photon annihilation (creation) operator and σ_z and σ_x are Pauli operators describing the qubit state. The effective charge-photon coupling strength $g = g_0 \sin\theta$ is normalized by the mixing angle θ, where $\sin\theta = 2t_{\text{c}}/\sqrt{\delta^2 + 4t_{\text{c}}^2}$ and g_0 is the charge-photon coupling strength at $\delta = 0$ (*23*). Applying the rotating wave approximation (RWA) in the frame rotating with the drive frequency $\omega_{\text{d}} = 2\pi f_{\text{d}}$ yields the typical Jaynes-Cummings (JC) Hamiltonian depicted in Eq. (S2), with the qubit lowering (raising) operator σ_- (σ_+). Note that the RWA eliminates the counter-rotating terms proportional to $a\sigma_-$ and $a^\dagger\sigma_+$.

$$H_{\text{JC}}/\hbar = \Delta_{\text{c}} a^\dagger a + \frac{\Delta_{\text{q}}}{2}\sigma_z + g(a^\dagger\sigma_- + a\sigma_+), \tag{S2}$$

where $\Delta_{\text{c}} = \omega_{\text{d}} - \omega_{\text{c}}$ and $\Delta_{\text{q}} = \omega_{\text{d}} - \omega_{\text{q}}$. We solve Eq. (S1) and Eq. (S2) to obtain the eigenspectrum shown in full and dashed lines respectively in Figs. 2C and D, using the parameters reported in Table S1.

In general, the input-output relation that describes a reflection-type cavity coupled to a qubit is written as

$$S_{11} = \frac{\Delta_c + i(\kappa_c - \kappa_i)/2 + g\chi}{\Delta_c + i\kappa/2 + g\chi}, \tag{S3}$$

where χ is the susceptibility of the qubit to the cavity photon, κ_c is the cavity-feedline coupling rate, κ_i is the internal cavity loss and $\kappa = \kappa_c + \kappa_i$ is the total cavity loss rate. The two Hamiltonians of Eq. (S2) and Eq. (S1) yield the distinct susceptibilities Eq. (S4) (*25*) and Eq. (S5) (*62*), respectively:

$$\chi_{JC} = \frac{g}{-\Delta_q - i\Gamma_{tot}} \tag{S4}$$

$$\chi_{full} = \frac{g}{-\Delta_q - i\Gamma_{tot}} + \frac{g}{-(\omega_d + \omega_q) - i\Gamma_{tot}}, \tag{S5}$$

where Γ_{tot} represents the decoherence rate of the qubit (*25*). For a hybrid system with strong coupling between the subsystems, i.e. with g/ω_c approaching 0.1 or higher, the counter-rotating terms become non-negligible, and the JC model fails to describe the system (*42, 43, 60*).

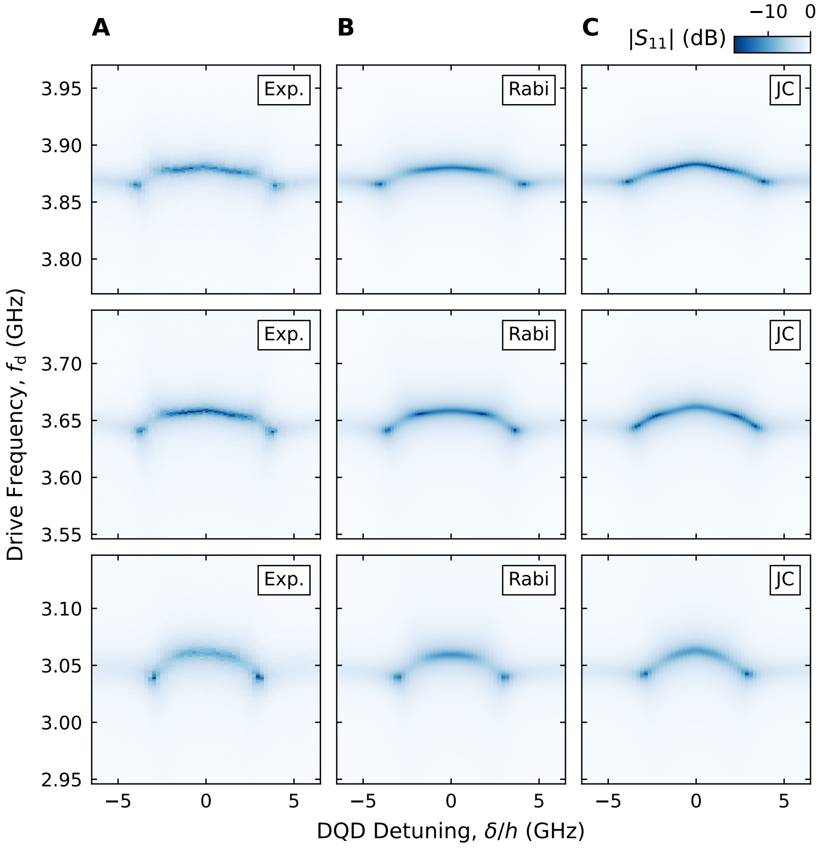

Figure S1: **Comparison of different fitting models.** (**A**) Measured normalized cavity reflectance $|S_{11}|$ as a function of the cavity drive frequency f_d and DQD detuning δ for different cavity resonance frequencies f_c = 3.878, 3.646, and 3.032 GHz. Simulated $|S_{11}|$ for each f_c using an input-output model built with (**B**) full-Rabi Hamiltonian taking into account the counter-rotating terms and (**C**) Jaynes-Cummings Hamiltonian neglecting the counter-rotating terms.

This can be observed in our system, where $g/\omega_q \sim 0.1 - 0.12$ at $\delta = 0$. In Fig. S1A, we show a single tone spectroscopy measurement of the DQD configuration shown in Fig. 2 for three different cavity resonance frequencies $f_c = 3.032$, 3.646 and 3.872 GHz. We numerically fit the input-output model Eq. (S3) to the spectra with $\chi = \chi_{\text{full}}$ to yield the simulated spectra in Fig. S1B. We also show the cavity spectra simulated by using the input-output model with $\chi = \chi_{\text{JC}}$ in Fig. S1C, which deviates from the experimental data in Fig. S1A, not properly capturing the Bloch-Siegert shift around $\delta = 0$ (*42*). The estimated device parameters of the photodetector shown in Fig. 2 and Fig. 3 are reported in Table S1. g_0, t_c and Γ_{tot} have been estimated using the full input-ouput model with χ_{full}.

f_c	3032 MHz	3646 MHz	3872 MHz
$\kappa/2\pi$	28.6 ± 0.3	28.1 ± 0.3	25.1 ± 0.3
$\kappa_c/2\pi$	22.8 ± 0.2	23.0 ± 0.2	18.3 ± 0.1
$\kappa^+_{\text{DQD}}/2\pi$	28.1 ± 2.5	27.3 ± 1.5	24.1 ± 1.2
$\kappa^-_{\text{DQD}}/2\pi$	29.3 ± 1.7	32.1 ± 1.3	26.7 ± 1.2
$g_0/2\pi$	183.7 ± 0.5	213.7 ± 0.3	210.6 ± 0.5
t_c/h	878.0 ± 4.4	878.0 ± 4.4	878.0 ± 4.4
$\Gamma_{\text{tot}}/2\pi$	722.2 ± 5.4	829.3 ± 3.6	893.0 ± 6.3
η^+ (%)	22.1 ± 1.9	55.8 ± 4.0	37.0 ± 2.5
η^- (%)	26.9 ± 2.4	67.7 ± 4.8	44.3 ± 3.0

Table S1: Device parameters from fits at three different cavity resonance frequencies f_c. κ and κ_c are extracted from bare cavity spectroscopy measurements, while g_0, t_c and Γ_{tot} are obtained from the fits reported in Fig. S1B using an input-output model that retains the counter-rotating terms. κ_{DQD} and η are extracted from the fits reported in Figs. 3E and B, respectively, for $\delta = \pm\delta_r$. The uncertainty of η is given by the uncertainty of the linear fit to Eq. (S9) and by propagating the fitting uncertainty of the attenuation factor β (see Supplementary Text S6 for more details). All values except for η are reported in MHz.

S3 Measurement Setup

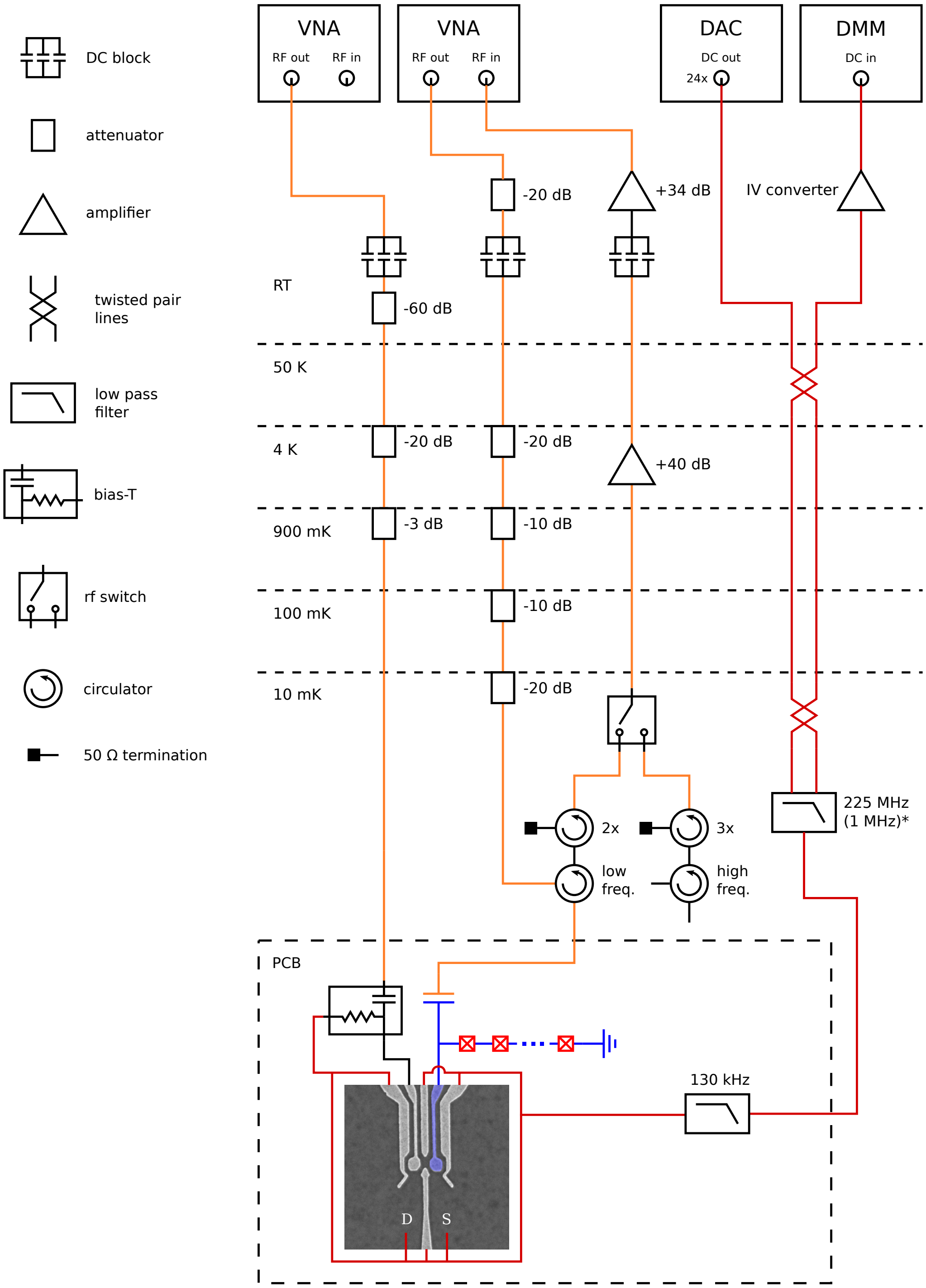

Figure S2: **Schematic of the cryogenic measurement setup.** The orange lines denote the coaxial cables hosting rf signals, while the red lines denote the twisted pair dc cables.

The measurements reported in this work are performed in a bottom loading dilution refrigerator (Bluefors LD250) at base temperatures around 10 mK (see Fig. S2). The device is mounted on a

printed circuit board (PCB, QDevil QBoard II), which consists of a daughterboard hosting the device and a motherboard, to which the daughterboard is connected via spring contacts. The motherboard features an RC low pass filtering stage (130 kHz cut-off) as well as bias-Ts for combining rf and dc signals. The PCB is mounted inside a fast sample exchange (FSE) probe ($T \sim 50$ mK), which is inserted into the cryostat without warming up the full system.

Gate and bias voltages for the QDs are generated by a 24-channel digital-to-analog converter (DAC, QDevil QDAC-II) and are passed to the PCB via twisted pair cables made of phosphor bronze and an additional LC low-pass filter stage at the mixing chamber plate at $T \sim 10$ mK. For the dc connections of the DQD gate lines, two different kinds of LC filters are used, a QDevil filter box (225 MHz cut-off) and a MFT25-150ohm box (1 MHz cut-off) by Basel Precision Instruments. The drain current through the device is measured by a digital multimeter (DMM, Keysight 34465A) after being amplified by an IV converter (Basel Precision Instruments SP 983C). The in-plane magnetic field is applied by a 6-1-1 T vector magnet by American Magnetics.

Spectroscopy measurements are performed with a vector network analyzer (VNA, Rohde & Schwarz ZNB20). The VNA output is attenuated at room temperature, followed by a dc block (Inmet 8039, inner-outer, 10 MHz cut-off), followed by another attenuation chain in the cryostat (-60 dB). The dc block is essential to avoid ground loops that would introduce low-frequency noise in the DQD gate lines or ohmic contacts. To measure the signal reflected by the cavity in a wide frequency range, we use two different sets of circulators, a triple junction low-frequency circulator (Quinstar QCY-G0250403AM, 2.5 – 4 GHz) and two double junction high-frequency circulators (Low Noise Factory CICIC4_12A, 4 – 12 GHz). A microwave switch (Radiall R570F32000) allows to choose which set of circulators to connect to the broadband HEMT amplifier at 4 K (Low Noise Factory LNC0.3_14B, 0.3 – 14 GHz, +40 dB). After amplification at 4 K, the reflected signal passes through another dc block (Inmet 8039) and a low noise amplifier at room temperature (Agile AMT_A0253, 0.1 – 20 GHz, +34 dB). For two-tone spectroscopy measurements, the second tone is applied by another VNA (Rohde & Schwarz ZNB20) in a fixed frequency mode. The DQD drive line, with a total cryogenic attenuation of 23 dB, is connected to the left plunger gate (V_{pL} in Fig. 1B) of the DQD via bias-T on the PCB. Contrary to the cavity drive line, for the qubit drive line the attenuators at room temperature are placed in between dc block and the cryostat to attenuate standing waves between the dc block and the bias-T.

S4 Noise-Induced Currents in Magnetic Field

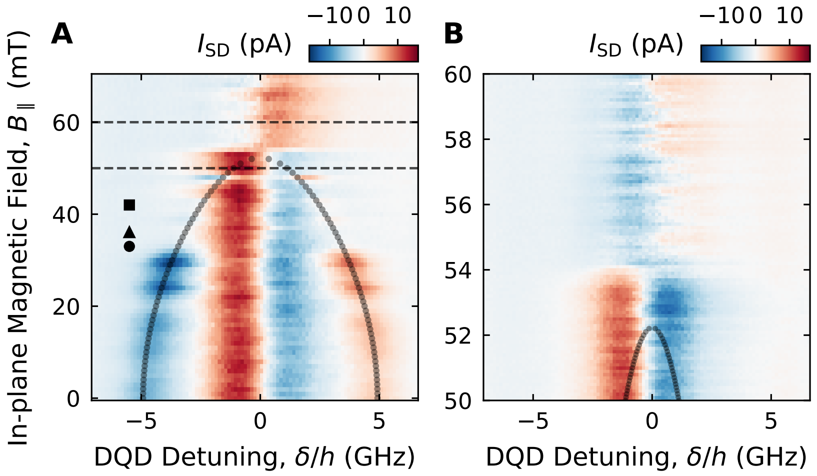

Figure S3: **Magnetic-field dependence of the dark current.** (**A**) DQD source-drain current I_{SD} as a function of the in-plane magnetic field $B_{\|}$ parallel to the JJ array (see Fig. 1C) and the DQD detuning δ in the same region as in Fig. 3, without any cavity drive. The grey dots denote the magnetic field for which qubit and cavity are in resonance. Black square, triangle and circle illustrate the three different $B_{\|}$ at which we extensively investigated the photon detection, also reported in Fig. S1. (**B**) Zoom-in of the region enclosed by the horizontal dashed lines in (**A**).

As evident from the charge stability diagram shown in Fig. 2A, a finite dark current is present near $\delta \sim 0$, even with negligibly small excitation in the cavity ($n_c \ll 1$). The dark current shows an antisymmetric behavior as a function of δ, which indicates the presence of detuning-dependent transport processes enabled by by phonons (*63*) or electrical noise (*40*) in the setup. While pinpointing the exact origin of these dark currents is out of the scope of this work, we show that they are enhanced by the superconducting environment by measuring I_{SD} around $\delta = 0$ as a function of the in-plane magnetic field $B_{\|}$. Figure S3A shows the same current around $\delta = 0$ as Fig. 2A, but as we increase the magnetic field the nature of the current changes. A zoom-in at higher magnetic fields (see Fig. S3B) reveals that this current decreases and even reverses polarity around 54 mT, consistent with the expected critical field of the superconducting Al layers. One possible explanation for this modification of the dark current is that spurious electromagnetic modes in the superconducting environment generate fluctuations in the DQD potential $\bar{\mu}$, thereby inducing a detuning-dependent current with polarity opposite to that of the PAT current. Once the Al becomes resistive, these fluctuations are strongly damped, leading to a pronounced suppression of the dark-current amplitude. The residual dark current observed above 54 mT in Fig. S3B appears to have a different origin, as indicated by another chang of polarity.

Another contribution to the dark current appears at detuning values $\delta = \pm\delta_r$ (indicated by grey dots in Figs. S3A and B), where the DQD charge qubit is resonant with the cavity. This feature indicates that the current originates from a residual photon population in the cavity. The strong variation of its amplitude as a function of magnetic field (and thus cavity frequency) suggests that this population is driven by frequency-dependent slot modes in the chip (see Fig. 1D). We stress, that the characterization of the photodetector in the high-efficiency configuration reported in

Fig. 3 and Table S1 is performed at frequencies for which this noise-induced current is suppressed (indicated by black square, triangle and circle in Fig. S3A). This explains the comparably low dark current observed at finite detuning in the reported measurements. Because the exact origin of the remaining dark current is difficult to identify, it cannot be straightforwardly associated with a dark count rate, i.e., a rate of false detection events. This is particularly true when some of the underlying mechanisms, as discussed above, produce currents with opposite polarity.

S5 Alternative DQD Configuration

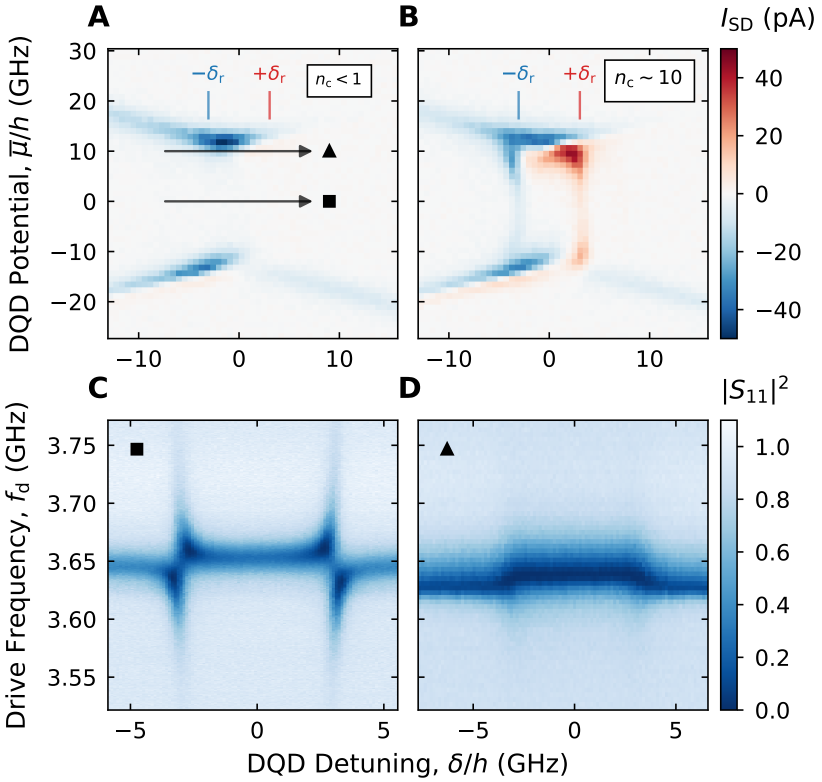

Figure S4: **Alternative DQD configuration to the one shown in Fig. 2 with reduced cotunneling current.** DQD charge stability diagrams measured as a function of the DQD detuning δ and potential $\bar{\mu}$ while monitoring the DQD source-drain current I_{SD} with (**A**) low ($n_c < 1$) and (**B**) high ($n_c \sim 10$) cavity drive power. $\pm\delta_r$ denote the DQD detuning values at which the charge qubit energy matches that of the cavity photon. In panel B, clear features related to photon-assisted tunneling appear at $\pm\delta_r$. (**C**-**D**) Normalized cavity reflectance $|S_{11}|$ measured as a function of the drive frequency f_d while sweeping δ along the arrow denoted by (**C**) square ($\bar{\mu}/h \sim 0$ GHz) and (**D**) triangle ($\bar{\mu}/h \sim 10$ GHz) in panel A.

A different DQD configuration with respect to the one presented in Fig. 2 has been studied and is presented in Fig. S4. This configuration does not feature any antisymmetric current around $\delta = 0$ at low cavity drive power, in contrast to the previous DQD configuration (see Fig. S4A). At higher drive power ($n_c \sim 10$), the stability diagram exhibits a strong PAT current symmetrically around the charge triple points (see Fig. S4B). In contrast to the configuration in Fig. 2, this PAT

current is significantly reduced when going toward the center of the inter-dot transition ($\bar{\mu} = 0$), which means that cotunneling effects are less prominent in this configuration. This observation is also reflected in the spectroscopy measurements taken along the black arrows in Fig. S4A. Close to the charge triple point (Fig. S4D, triangle cut from Fig. S4A), the charge qubit decoherence rate is significantly larger than the one at $\bar{\mu} = 0$ (Fig. S4C, square cut from Fig. S4A), due to the large relaxation rate to the reservoirs. The different behavior compared to Fig. 2 likely stems from a combination of smaller tunneling rate to the reservoirs and larger inter-dot charging energy U, which reduces cotunneling effects (*46*).

S6 ac Stark Shift Measurements

In the dispersive regime, i.e. when the detuning between qubit and cavity $\Delta_{qc} = \omega_q - \omega_c$ is large enough, such that $\Delta_{qc} \gg g$, the Jaynes-Cummings Hamiltonian in Eq. (S2) can be reduced to

$$H = \hbar\omega_c a^\dagger a + \frac{1}{2}\hbar(\omega_q + \chi + 2\chi a^\dagger a)\sigma_z, \tag{S6}$$

where $\chi = g^2/\Delta_{qc}$ is the dispersive shift (*33*). We can thus write the dressed qubit frequency as $\tilde{\omega}_q = \omega_q + \chi + 2n_c\chi$, with the average cavity photon number $n_c = \langle a^\dagger a \rangle$. The coupling to the cavity therefore leads to a power-dependent ac Stark shift of

$$\Delta\nu = (\tilde{\omega}_q - \tilde{\omega}_{q,0})/2\pi = 2n_c g^2/(2\pi\Delta_{qc}), \tag{S7}$$

where $\tilde{\omega}_{q,0}$ is the measured qubit frequency at low cavity drive power ($n_c \ll 1$). The average photon number can be related to the input power P_d using

$$n_c = \frac{4\kappa_c}{\kappa^2}\dot{N} = \frac{4\kappa_c}{\kappa^2}\frac{P_d}{hf_c} = \frac{4\kappa_c}{\kappa^2}\frac{\beta P_{VNA}}{hf_c}, \tag{S8}$$

where P_{VNA} is the VNA output power and $\beta = P_d/P_{VNA}$ is the total attenuation factor taking into account all the losses between the VNA and the cavity feedline. Therefore, measuring the ac Stark shift in a stable DQD configuration is a useful tool for estimating the effective power reaching the device at a specific cavity frequency (*33*). Combining $\beta = P_d/P_{VNA}$ with the relation $\dot{N} = P_d/hf_c$ then allows one to extract the charge–photon conversion efficiency $\eta = I_{SD}/e\dot{N}$ as

$$\eta = \frac{I_{SD}}{P_{VNA}}\frac{hf_c}{e}\frac{1}{\beta}. \tag{S9}$$

In a separate cooldown from the one in Fig. 2 and Fig. 3, we tune the device to a more coherent DQD charge configuration (shown in Fig. S5A) obtained by defining large mutual capacitance between the two QDs to reduce its sensitivity to charge noise (*34*) and drastically reducing the tunneling rate to the QD reservoirs. In Fig. S5B, we present a single tone spectroscopy measurement, implemented as in Fig. 2C, as a function of δ measured along the black arrow in Fig. S5A. A clear vacuum-Rabi splitting at finite δ indicates that the qubit frequency lies well below f_c near $\delta = 0$. To accurately estimate the qubit frequency, we perform two-tone spectroscopy (*33, 64*) (shown in Fig. S5C) by applying a qubit pump tone at frequency f_p to the left plunger gate (see Supplementary Text S3), while measuring the cavity at its resonance frequency $f_d = f_c$. We fit a Lorentzian curve

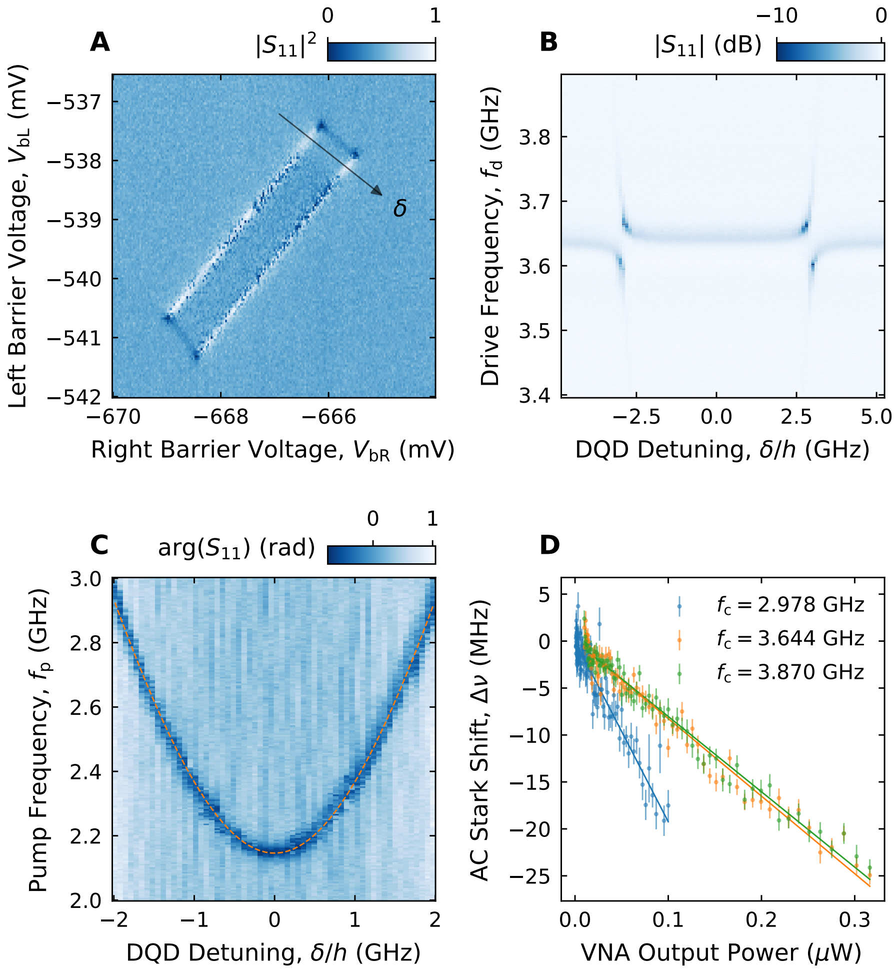

Figure S5: **ac Stark shift measurements for input loss characterization.** (**A**) DQD charge stability diagram of a new configuration in the same device, measured as a function of the right and left barrier gate voltage V_{bR} and V_{bL} (see Fig. 1B) by monitoring the normalized cavity reflectance $|S_{11}|^2$. (**B**) $|S_{11}|$ as a function of the drive frequency f_{d} and DQD detuning δ tuned along the black arrow in panel (**A**). (**C**) DQD charge qubit excitation frequency measured by monitoring the phase of the cavity reflectance $\arg(S_{11})$ as a function of δ and the qubit pump frequency f_{p}. The cavity drive tone is kept at $f_{\mathrm{d}} = f_{\mathrm{c}} \sim 3.644$ GHz. The dashed orange curve represents a numerical fit to the charge qubit frequency $f_{\mathrm{q}} = \sqrt{\delta^2 + 4t_{\mathrm{c}}^2}/h$, from which we extract $2t_{\mathrm{c}}/h \sim 2.2$ GHz. (**D**) ac Stark shift $\Delta\nu$ of the qubit measured by recording f_{q} at $\delta = 0$ as a function of the VNA output power applied to the cavity at $f_{\mathrm{d}} = f_{\mathrm{c}} \sim 2.978$ GHz (blue dots), 3.644 GHz (orange dots) and 3.870 GHz (green dots). The solid lines represent linear fits to Eq. (S7) from which we extract the input loss factors β for the frequencies in the legend.

to the spectrum at each detuning value to extract f_{q} and fit it to the expression $f_{\mathrm{q}} = \sqrt{\delta^2 + 4t_{\mathrm{c}}^2}/h$ yielding $t_{\mathrm{c}}/h = 1073 \pm 6.7$ MHz. With this value of t_{c}, we fit the input-output model described in Supplementary Text S2 to the single tone spectroscopy data shown in Fig. S5B extracting a coupling strength of $g_0/2\pi = 108.9 \pm 2.7$ MHz.

We then measure the ac Stark shift of the charge qubit at $\delta = 0$ as a function of P_{VNA} for three cavity resonance frequencies close to the ones used in Fig. S1. The dots in Fig. S5D represent the measured qubit frequency shifts $\Delta\nu$ and the solid lines are linear fits according to Eq. (S7), from which we get the total attenuation factor β. The extracted parameters for all three cavity frequencies are reported in Table S2. For the measurements at $f_c = 2.978$ GHz, a different DQD configuration was used compared to $f_c = 3.644$ GHz and $f_c = 3.870$ GHz. The same procedure is performed with the high-frequency setup in order to characterize the input loss for the photon detection measurements at high frequencies (red dots in Fig. 4A).

f_c	2978 MHz	3644 MHz	3870 MHz
$\kappa/2\pi$	23.9 ± 0.2	26.4 ± 0.3	21.4 ± 0.2
$\kappa_c/2\pi$	18.2 ± 0.1	22.2 ± 0.2	15.3 ± 0.1
$g_0/2\pi$	85.8 ± 1.8	108.9 ± 2.7	112.3 ± 3.0
t_c/h	891.1 ± 9.4	1073.1 ± 6.7	1073.1 ± 6.7
$\beta \cdot 10^9$	1.52 ± 0.13	0.62 ± 0.04	0.66 ± 0.05

Table S2: Device parameters used for characterizing the input loss at the frequencies close to where the photon detection performance has been studied in the main text. κ and κ_c are obtained from bare cavity spectroscopy measurements, g_0 (t_c) is extracted from the single tone (two-tone) spectroscopy measurement reported in Fig. S5B (Fig. S5C). The total loss factor β is extracted from the linear fit shown in Fig. S5D using to Eq. (S7) and Eq. (S8). The uncertainty of β is obtained from the uncertainty of the linear fit as well as the propagation of the fitting uncertainties of the other parameters in this table. The dominating uncertainties stem from the estimation of the slope and the fitting uncertainty of g_0. All values except for the dimensionless β are reported in MHz.

S7 Cavity Characterization

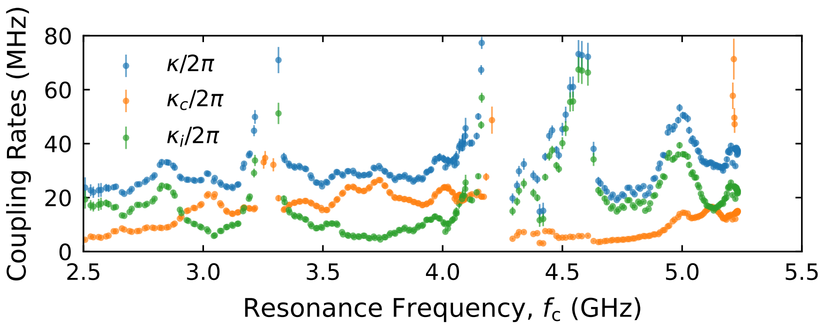

Figure S6: **Cavity coupling rates as a function of cavity resonance frequency f_c.** Obtained from fitting the cavity magnetospectroscopy in Fig. 1D (for $f_c < 4.2$ GHz) and another similar measurement (for $f_c > 4.2$ GHz) using the low- and high-frequency setup, respectively (see Fig. S2 in Supplementary Text S3).

The bare cavity is characterized as a function of the cavity resonance frequency f_c with the DQD in Coulomb blockade, ensuring no interaction with the cavity. The cavity-feedline coupling rate κ_c, internal loss rate κ_i, and total cavity loss rate κ presented in Fig. S6 are estimated by fitting the input-output model from Eq.(S3) to magnetospectroscopy measurements with a low cavity drive power P_d ($n_\mathrm{c} < 1$). For $f_\mathrm{c} < 4.2$ GHz, the data reported in Fig. 1D, measured with the low-frequency circulator setup (described in Fig. S2 in Supplementary Text S3), is used, whereas the values for $f_\mathrm{c} > 4.2$ GHz are extracted from another measurement using the high-frequency circulator setup.

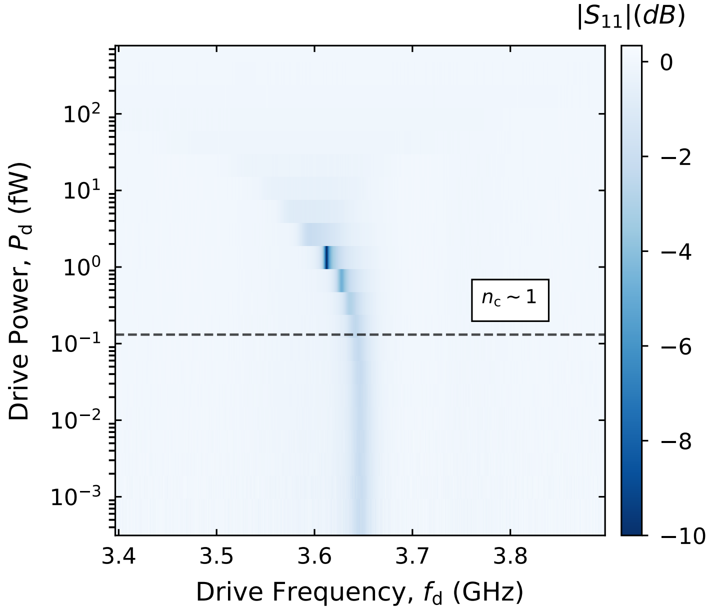

Figure S7: **Power dependence of the cavity response.** Normalized cavity reflectance $|S_{11}|^2$ as a function of the cavity drive frequency f_d and the feedline drive power P_d with the cavity resonance frequency $f_\mathrm{c} \sim 3.646$ GHz. The power for which the average cavity photon number $n_\mathrm{c} \sim 1$ is indicated by a black dashed line. For higher drive power ($n_\mathrm{c} > 1$) the resonance dip shifts to lower frequencies due to the self-Kerr non-linearity of the JJ array cavity (*65*).

The power dependence of the cavity response is studied by measuring the normalized cavity reflectance $|S_{11}|^2$ as a function of f_d and P_d, and is reported in Fig. S7. The self-Kerr nonlinearity, arising from the intrinsic nonlinearity of the Josephson junctions, leads to a power-dependent shift of f_c (*65*). Furthermore, the average cavity photon number n_c exhibits a nonlinear dependence on P_d at higher powers (*49*). These nonlinear effects do not impact the photodetector characterization carried out for $n_\mathrm{c} < 1$ (below the dashed line in Fig. S7).

S8 Impact of System Parameters on Efficiency

Figure S8 demonstrates the microwave photon detection efficiency η as a function of various parameters in the hybrid system. In the following analysis, we assume that $\Gamma_{0\mathrm{e}}/2\pi$ is fully tunable within the range 0 – 4 GHz to match $\kappa_\mathrm{DQD} = 4g^2/\Gamma_\mathrm{tot} = \kappa$ with $\Gamma_\mathrm{tot} = \Gamma_{0\mathrm{e}} + \gamma_- + 2\gamma_\phi$ at each point, to ensure $\frac{4\kappa_\mathrm{DQD}\kappa}{(\kappa_\mathrm{DQD}+\kappa)^2} = 1$ (Eq. (1)). In Figs. S8A and C η is shown as a function of the

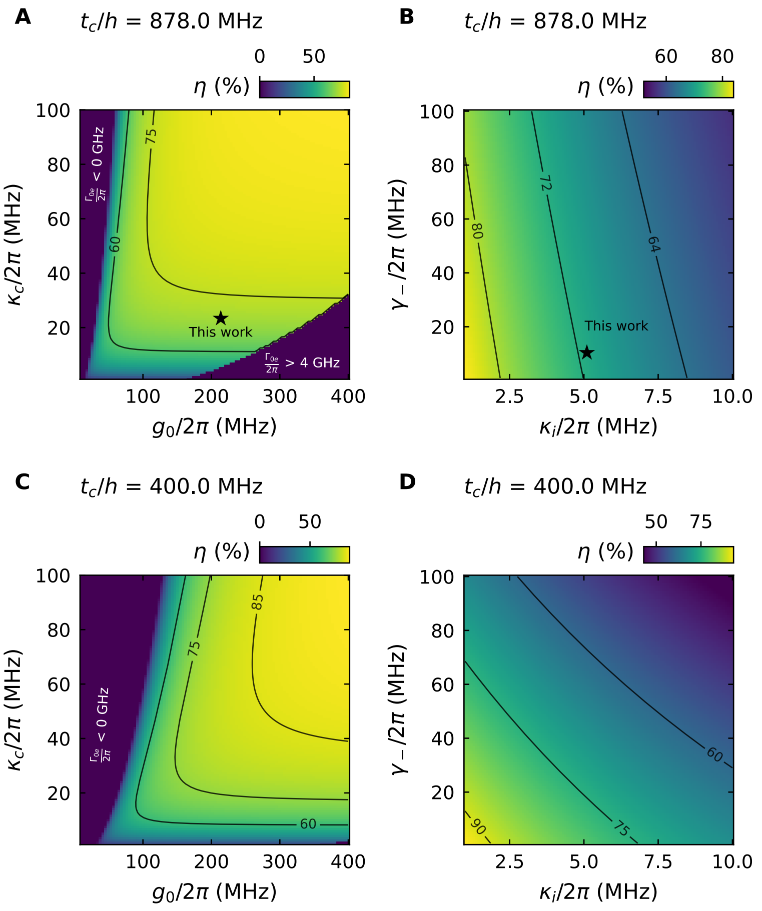

Figure S8: **Dependence of the microwave photon detection efficiency η on various system parameters.** η is calculated as a function of (**A**), (**C**) κ_c and g_0, and (**B**), (**C**) γ_- and κ_i. For panels (**A**) and (**B**), we use an inter-dot tunnel coupling strength of $t_c/h \sim 878$ MHz, as extracted from the configuration shown in Fig. 2, whereas, for panels (**C**) and (**D**), we assume $t_c/h = 400$ MHz. The relaxation rate of the qubit excited state to the reservoirs $\Gamma_{0e}/2\pi$ is assumed to be tunable from 0–4 GHz to enforce $\kappa_{DQD} = \kappa$ in all simulations. The remaining system parameters, including κ_i and g_0, are assumed to be identical to the ones of the system configuration in Fig. 2 and Fig. 3.

cavity coupling strength κ_c and charge-photon coupling strength g_0. We also show η as a function of the inter-dot relaxation rate γ_- and internal cavity loss rate κ_i in Figs. S8B and D. For the analysis presented in Figs. S8A and B, we use parameter values identical to those extracted from the experiments at the cavity resonance frequency $f_c = 3.646$ GHz ($\kappa/2\pi = 28.1$ MHz, $\kappa_c/2\pi = 23.0$ MHz, $g_0/2\pi = 213.7$ MHz, $t_c/h = 878.0$ MHz), and assume symmetric QD-reservoir tunneling rates ($\Gamma_L = \Gamma_R$). For the inter-dot relaxation rate γ_- and dephasing rate γ_ϕ, which cannot be individually assessed from the experiments, we empirically assume $\gamma_-/2\pi = \gamma_\phi/2\pi = 10$ MHz. These parameters yield the calculated $\eta \sim 71\%$ which is close to the measured $\eta \sim 67.7\%$ at $f_c = 3.646$ GHz.

As clearly demonstrated by Fig. S8A, a large g_0 allows high η in a wide parameter window.

Combined with the large tunability of the DQD tunneling rates, this also makes the photon detection more robust to non-uniformity and disorder and less sensitive to fabrication variability. Also, simply increasing κ_c by changing the device geometry, while keeping the other parameters constant, will allow η to reach $\sim$ 80 %. Alternatively as demonstrated in Fig. S8B, decreasing the loss rate of each subsystem may also lead to $\eta > 80$ %. Figures S8C and D present calculations similar to the ones shown in Figs. S8A and B, but with smaller $t_c/h = 400$ MHz while keeping the other parameters identical. This results in a larger directivity D to yield higher η, where Fig. S8D explicitly demonstrates that further optimization of the system loss rates in addition to the smaller t_c can result in $\eta > 90\%$.

S9 Comparison to previous DQD-cavity implementations

	Ref. (*27*)	Ref. (*32*)	This work
f_c	6436	6716	3646
$\kappa/2\pi$	15.5	5.2	28.1
$\kappa_c/2\pi$	4.3	3.9	23
$\kappa_{DQD}/2\pi$	6.9	0.8	32.1
$g_0/2\pi$	38	43	214
t_c/h	1959	500	878
$\Gamma_{tot}/2\pi$	790	2600	829
$\Gamma_{0e}/2\pi$	46	2157	> 486
$\gamma_-/2\pi$	23	13	-
η	0.06	0.25	0.68
κ_c/κ	0.278	0.750	0.819
$\frac{4\kappa_{DQD}\kappa}{(\kappa_{DQD}+\kappa)^2}$	0.853	0.462	0.996
$\frac{\Gamma_{0e}}{\Gamma_{0e}+\gamma_-}$	0.667	0.994	0.952
D	0.793	0.989	0.876

Table S3: Comparison of the estimated detector parameters of previous implementations of QD-based microwave photodetectors with this work. The system parameters are reported in units MHz, while the efficiency η and the terms affecting it (according to Eq. (1)) are dimensionless.

Table S3 compares the estimated system parameters of the detector presented in this work with those reported in previous implementations in Refs. (*27, 32*). Although the performance of our device remains mostly limited by the ratio κ_c/κ, it still exhibits the highest ratio among the three implementations listed in Table S3. In Ref. (*27*), the reduced κ_c/κ ratio is primarily attributed to the transmission-style geometry used for cavity coupling. Another significant advantage of our detector is the tunability of both g and Γ_{0e}, which enables simultaneous optimization of $4\kappa_{DQD}\kappa/(\kappa_{DQD}+\kappa)^2$ and $\Gamma_{0e}/(\Gamma_{0e}+\gamma_-)$. Achieving this level of simultaneous control over both quantities was difficult in Refs. (*27, 32*).

REFERENCES

1. R. H. Hadfield, Single-photon detectors for optical quantum information applications. *Nat. Photonics* **3**, 696–705 (2009.

2. N. Gisin, G. Ribordy, W. Tittel, H. Zbinden, Quantum cryptography. *Rev. Mod. Phys.* **74**, 145–195 (2002).

3. J. L. O'Brien, Optical quantum computing. *Science* **318**, 1567–1570 (2007).

4. R. H. Hadfield, J. Leach, F. Fleming, D. J. Paul, C. H. Tan, J. S. Ng, R. K. Henderson, G. S. Buller, Single-photon detection for long-range imaging and sensing. *Optica* **10**, 1124–1141 (2023).

5. M. D. Eisaman, J. Fan, A. Migdall, S. V. Polyakov, Invited review article: Single-photon sources and detectors. *Rev. Sci. Instrum.* **82**, 071101 (2011).

6. S. Cova, A. Longoni, A. Andreoni, Towards picosecond resolution with single-photon avalanche diodes. *Rev. Sci. Instrum.* **52**, 408–412 (1981).

7. H. Dautet, P. Deschamps, B. Dion, A. D. MacGregor, D. MacSween, R. J. McIntyre, C. Trottier, P. P. Webb, Photon counting techniques with silicon avalanche photodiodes. *Appl. Optics* **32**, 3894–3900 (1993).

8. H. Takesue, S. W. Nam, Q. Zhang, R. H. Hadfield, T. Honjo, K. Tamaki, Y. Yamamoto, Quantum key distribution over a 40-dB channel loss using superconducting single-photon detectors. *Nat. Photonics* **1**, 343–348 (2007).

9. X. Gu, A. F. Kockum, A. Miranowicz, Y.-x. Liu, F. Nori, Microwave photonics with superconducting quantum circuits. *Phys. Rep.* **718-719**, 1–102 (2017).

10. S. R. Sathyamoorthy, T. M. Stace, G. Johansson, Detecting itinerant single microwave photons. *Comptes Rendus. Physique* **17**, 756–765 (2016).

11. L. Balembois, J. Travesedo, L. Pallegoix, A. May, E. Billaud, M. Villiers, D. Esteve, D. Vion, P. Bertet, E. Flurin, Cyclically operated microwave single-photon counter with sensitivity of $10^{-22}\,\mathrm{W}/\sqrt{\mathrm{Hz}}$. *Phys. Rev. Appl.* **21**, 014043 (2024).

12. J.-C. Besse, S. Gasparinetti, M. C. Collodo, T. Walter, P. Kurpiers, M. Pechal, C. Eichler, A. Wallraff, Single-shot quantum nondemolition detection of individual itinerant microwave photons. *Phys. Rev. X* **8**, 021003 (2018).

13. K. Inomata, Z. Lin, K. Koshino, W. D. Oliver, J. S. Tsai, T. Yamamoto, Y. Nakamura, Single microwave-photon detector using an artificial Λ-type three-level system. *Nat. Commun.* **7**, 12303 (2016).

14. Y.-F. Chen, D. Hover, S. Sendelbach, L. Maurer, S. T. Merkel, E. J. Pritchett, F. K. Wilhelm, R. McDermott, Microwave photon counter based on josephson junctions. *Phys. Rev. Lett.* **107**, 217401 (2011).

15. O. Stanisavljević, J. C. Philippe, J. Gabelli, M. Aprili, J. Estève, J. Basset, Efficient microwave photon-to-electron conversion in a high-impedance quantum circuit. *Phys. Rev. Lett.* **133**, 076302 (2024).

16. A. L. Pankratov, A. V. Gordeeva, A. V. Chiginev, L. S. Revin, A. V. Blagodatkin, N. Crescini, L. S. Kuzmin, Detection of single-mode thermal microwave photons using an underdamped Josephson junction. *Nat. Commun.* **16**, 3457 (2025).

17. Y. Q. Chai, S. N. Wang, P. H. OuYang, L. F. Wei, Measuring weak microwave signals via current-biased Josephson junctions: Approaching the quantum limit of energy detection. *Phys. Rev. B* **111**, 024501 (2025).

18. J. Basset, O. Stanisavljević, J. Gabelli, M. Aprili, J. Estève, Fast and continuous detection of single microwave photons via photo-assisted quasiparticle tunneling to a superconducting island. arXiv:2511.17470 [cond-mat.mes-hall] (2025).

19. K. Petrovnin, J. Wang, M. Perelshtein, P. Hakonen, G. S. Paraoanu, Microwave photon detection at parametric criticality. *PRX Quantum* **5**, 020342 (2024).

20. G.-H. Lee, D. K. Efetov, W. Jung, L. Ranzani, E. D. Walsh, T. A. Ohki, T. Taniguchi, K. Watanabe, P. Kim, D. Englund, K. C. Fong, Graphene-based Josephson junction microwave bolometer. *Nature* **586**, 42–46 (2020).

21. R. Kokkoniemi, J. P. Girard, D. Hazra, A. Laitinen, J. Govenius, R. E. Lake, I. Sallinen, V. Vesterinen, M. Partanen, J. Y. Tan, K. W. Chan, K. Y. Tan, P. Hakonen, M. Möttönen, Bolometer operating at the threshold for circuit quantum electrodynamics. *Nature* **586**, 47–51 (2020).

22. Y.-C. Chang, F. Chianese, N. Shetty, J. Huhtasaari, A. Jayaraman, J. T. Peltonen, S. Lara-Avila, B. Karimi, A. Danilov, J. P. Pekola, S. Kubatkin, Quantum-ready microwave detection with scalable graphene bolometers in the strong localization regime. arXiv:2505.24564 [cond-mat.mes-hall] (2025).

23. A. Stockklauser, P. Scarlino, J. V. Koski, S. Gasparinetti, C. K. Andersen, C. Reichl, W. Wegscheider, T. Ihn, K. Ensslin, A. Wallraff, Strong coupling cavity QED with gate-defined double quantum dots enabled by a high impedance resonator. *Phys. Rev. X* **7**, 011030 (2017).

24. X. Mi, J. V. Cady, D. M. Zajac, P. W. Deelman, J. R. Petta, Strong coupling of a single electron in silicon to a microwave photon. *Science* **355**, 156–158 (2017).

25. F. De Palma, F. Oppliger, W. Jang, S. Bosco, M. Janík, S. Calcaterra, G. Katsaros, G. Isella, D. Loss, P. Scarlino, Strong hole-photon coupling in planar Ge for probing charge degree and strongly correlated states. *Nat. Commun.* **15**, 10177 (2024).

26. N. Samkharadze, G. Zheng, N. Kalhor, D. Brousse, A. Sammak, U. C. Mendes, A. Blais, G. Scappucci, L. M. K. Vandersypen, Strong spin-photon coupling in silicon. *Science* **359**, 1123–1127 (2018).

27. W. Khan, P. P. Potts, S. Lehmann, C. Thelander, K. A. Dick, P. Samuelsson, V. F. Maisi, Efficient and continuous microwave photoconversion in hybrid cavity-semiconductor nanowire double quantum dot diodes. *Nat. Commun.* **12**, 5130 (2021).

28. C. H. Wong, M. G. Vavilov, Quantum efficiency of a single microwave photon detector based on a semiconductor double quantum dot. *Phys. Rev. A* **95**, 012325 (2017).

29. S. Haldar, D. Barker, H. Havir, A. Ranni, S. Lehmann, K. A. Dick, V. F. Maisi, Continuous microwave photon counting by semiconductor-superconductor hybrids. *Phys. Rev. Lett.* **133**, 217001 (2024).

30. D. Zenelaj, P. Samuelsson, P. P. Potts, Wigner-function formalism for the detection of single microwave pulses in a resonator-coupled double quantum dot. *Phys. Rev. Res.* **7**, 013305 (2025).

31. A. Ghirri, S. Cornia, M. Affronte, Microwave photon detectors based on semiconducting double quantum dots. *Sensors* **20**, 4010 (2020).

32. S. Haldar, High-efficiency microwave photodetection by cavity-coupled double quantum dots with single-cavity-photon sensitivity. *Phys. Rev. Appl.* **24**, 044074 (2025).

33. D. I. Schuster, A. Wallraff, A. Blais, L. Frunzio, R. S. Huang, J. Majer, S. M. Girvin, R. J. Schoelkopf, ac Stark shift and dephasing of a superconducting qubit strongly coupled to a cavity field. *Phys. Rev. Lett.* **94**, 123602 (2005).

34. P. Scarlino, J. H. Ungerer, D. J. van Woerkom, M. Mancini, P. Stano, C. Müller, A. J. Landig, J. V. Koski, C. Reichl, W. Wegscheider, T. Ihn, K. Ensslin, A. Wallraff, In situ tuning of the electric-dipole strength of a double-dot charge qubit: Charge-noise protection and ultrastrong coupling. *Phys. Rev. X* **12**, 031004 (2022).

35. R. Kuzmin, N. Mehta, N. Grabon, V. E. Manucharyan, Tuning the inductance of Josephson junction arrays without SQUIDs. *Appl. Phys. Lett.* **123**, 182602 (2023).

36. S. Huang, B. Lienhard, G. Calusine, A. Vepsäläinen, J. Braumüller, D. K. Kim, A. J. Melville, B. M. Niedzielski, J. L. Yoder, B. Kannan, T. P. Orlando, S. Gustavsson, W. D. Oliver, Microwave package design for superconducting quantum processors. *PRX Quantum* **2**, 020306 (2021).

37. Z. Chen, A. Megrant, J. Kelly, R. Barends, J. Bochmann, Y. Chen, B. Chiaro, A. Dunsworth, E. Jeffrey, J. Y. Mutus, P. J. J. O'Malley, C. Neill, P. Roushan, D. Sank, A. Vainsencher, J. Wenner, T. C. White, A. N. Cleland, J. M. Martinis, Fabrication and characterization of aluminum airbridges for superconducting microwave circuits. *Appl. Phys. Lett.* **104**, 052602 (2014).

38. G. Ponchak, J. Papapolymerou, M. Tentzeris, Excitation of coupled slotline mode in finite-ground CPW with unequal ground-plane widths. *IEEE Trans. Microw. Theory Tech.* **53**, 713–717 (2005).

39. D. Taubert, D. Schuh, W. Wegscheider, S. Ludwig, Determination of energy scales in few-electron double quantum dots. *Rev. Sci. Instrum.* **82**, 123905 (2011).

40. O. Entin-Wohlman, D. Chowdhury, A. Aharony, S. Dattagupta, Heat currents in electronic junctions driven by telegraph noise. *Phys. Rev. B* **96**, 195435 (2017).

41. M. Scigliuzzo, "Effects of the environment on quantum systems: Decoherence, bound states and high impedance in superconducting circuits," thesis, Chalmers University of Technology (2021).

42. P. Forn-Díaz, J. Lisenfeld, D. Marcos, J. J. García-Ripoll, E. Solano, C. J. P. M. Harmans, J. E. Mooij, Observation of the Bloch-Siegert shift in a qubit-oscillator system in the ultrastrong coupling regime. *Phys. Rev. Lett.* **105**, 237001 (2010).

43. T. Niemczyk, F. Deppe, H. Huebl, E. P. Menzel, F. Hocke, M. J. Schwarz, J. J. Garcia-Ripoll, D. Zueco, T. Hümmer, E. Solano, A. Marx, R. Gross, Circuit quantum electrodynamics in the ultrastrong-coupling regime. *Nat. Phys.* **6**, 772–776 (2010).

44. M. Devoret, S. Girvin, R. Schoelkopf, Circuit-QED: How strong can the coupling between a Josephson junction atom and a transmission line resonator be? *Ann. Phys.* **519**, 767–779 (2007).

45. S. De Franceschi, S. Sasaki, J. M. Elzerman, W. G. van der Wiel, S. Tarucha, L. P. Kouwenhoven, Electron cotunneling in a semiconductor quantum dot. *Phys. Rev. Lett.* **86**, 878–881 (2001).

46. S. Amasha, A. J. Keller, I. G. Rau, A. Carmi, J. A. Katine, H. Shtrikman, Y. Oreg, D. Goldhaber-Gordon, Pseudospin-resolved transport spectroscopy of the kondo effect in a double quantum dot. *Phys. Rev. Lett.* **110**, 046604 (2013).

47. T. H. Oosterkamp, L. P. Kouwenhoven, A. E. A. Koolen, N. C. v. d. Vaart, C. J. P. M. Harmans, Photon-assisted tunnelling through a quantum dot. *Semicond. Sci. Technol.* **11**, 1512–1515 (1996).

48. T. Fujisawa, S. Tarucha, Photon assisted tunnelling in single and coupled quantum dot systems. *Superlattices Microstruct.* **21**, 247–254 (1997).

49. C. Eichler, A. Wallraff, Controlling the dynamic range of a Josephson parametric amplifier. *EPJ Quantum Technol.* **1**, 1–19 (2014).

50. D. Bozyigit, C. Lang, L. Steffen, J. M. Fink, C. Eichler, M. Baur, R. Bianchetti, P. J. Leek, S. Filipp, M. P. da Silva, A. Blais, A. Wallraff, Antibunching of microwave-frequency photons observed in correlation measurements using linear detectors. *Nat. Phys.* **7**, 154–158 (2011).

51. A. Keränen, Q. M. Chen, A. Gunyhó, P. Singh, J. Ma, V. Vesterinen, J. Govenius, M. Möttönen, Correlation measurement of propagating microwave photons at millikelvin. *Nat. Commun.* **16**, 3875 (2025).

52. I.-C. Hoi, T. Palomaki, J. Lindkvist, G. Johansson, P. Delsing, C. M. Wilson, Generation of nonclassical microwave states using an artificial atom in 1D open space. *Phys. Rev. Lett.* **108**, 263601 (2012).

53. C. Bergenfeldt, P. Samuelsson, B. Sothmann, C. Flindt, M. Büttiker, Hybrid microwave-cavity heat engine. *Phys. Rev. Lett.* **112**, 076803 (2014).

54. S. Haldar, D. Zenelaj, P. P. Potts, H. Havir, S. Lehmann, K. A. Dick, P. Samuelsson, V. F. Maisi, Microwave power harvesting using resonator-coupled double quantum dot photodiode. *Phys. Rev. B* **109**, L081403 (2024).

55. H. Havir, A. Cicovic, P. Glidic, S. Haldar, S. Lehmann, K. A. Dick, V. F. Maisi, Near-unity charge readout in a nonlinear resonator without matching. arXiv:2505.17709 [cond-mat.mes-hall] (2025).

56. H. Geng, M. Kiczynski, A. V. Timofeev, E. N. Osika, D. Keith, J. Rowlands, L. Kranz, R. Rahman, Y. Chung, J. G. Keizer, S. K. Gorman, M. Y. Simmons, High-fidelity sub-microsecond single-shot electron spin readout above 3.5 K. *Nat. Commun.* **16**, 3382 (2025).

57. D. Zenelaj, P. P. Potts, P. Samuelsson, Full counting statistics of the photocurrent through a double quantum dot embedded in a driven microwave resonator. *Phys. Rev. B* **106**, 205135 (2022).

58. A. Opremcak, I. V. Pechenezhskiy, C. Howington, B. G. Christensen, M. A. Beck, E. Leonard Jr, J. Suttle, C. Wilen, K. N. Nesterov, G. J. Ribeill, T. Thorbeck, F. Schlenker, M. G. Vavilov, B. L. T. Plourde, R. McDermott, Measurement of a superconducting qubit with a microwave photon counter. *Science* **361**, 1239–1242 (2018).

59. Z. Wang, L. Balembois, M. Rančić, E. Billaud, M. le Dantec, A. Ferrier, P. Goldner, S. Bertaina, T. Chanelière, D. Esteve, D. Vion, P. Bertet, E. Flurin, Single-electron spin resonance detection by microwave photon counting. *Nature* **619**, 276–281 (2023).

60. A. Frisk Kockum, A. Miranowicz, S. De Liberato, S. Savasta, F. Nori, Ultrastrong coupling between light and matter. *Nat. Rev. Phys.* **1**, 19–40 (2019).

61. A. Blais, A. L. Grimsmo, S. Girvin, A. Wallraff, Circuit quantum electrodynamics. *Rev. Mod. Phys.* **93**, 025005 (2021).

62. S. Kohler, Dispersive readout: Universal theory beyond the rotating-wave approximation. *Phys. Rev. A* **98**, 023849 (2018).

63. S. Dorsch, A. Svilans, M. Josefsson, B. Goldozian, M. Kumar, C. Thelander, A. Wacker, A. Burke, Heat driven transport in serial double quantum dot devices. *Nano Lett.* **21**, 988–994 (2021).

64. P. Scarlino, D. J. van Woerkom, A. Stockklauser, J. V. Koski, M. C. Collodo, S. Gasparinetti, C. Reichl, W. Wegscheider, T. Ihn, K. Ensslin, A. Wallraff, All-microwave control and dispersive readout of gate-defined quantum dot qubits in circuit quantum electrodynamics. *Phys. Rev. Lett.* **122**, 206802 (2019).

65. Y. Krupko, V. D. Nguyen, T. Weißl, É. Dumur, J. Puertas, R. Dassonneville, C. Naud, F. W. J. Hekking, D. M. Basko, O. Buisson, N. Roch, W. Hasch-Guichard, Kerr nonlinearity in a superconducting Josephson metamaterial. *Phys. Rev. B* **98**, 094516 (2018).
